# Comparative assessment of seven surgical procedures in Carpal Tunnel Syndrome: a network meta-analysis empowering physician-patient decision-making

**DOI:** 10.1007/s10143-025-03587-x

**Published:** 2025-06-05

**Authors:** Amr Elrosasy, Mahmoud Diaa Hindawi, Qasi Najah, Mohamed Abo Zeid, Hatem Eldeeb, Asem Ahmed Ghalwash, Eslam Afifi, Abdallah Bani-Salameh, Nereen Almosilhy, Mohamed Ahmed Shahen, Fatma Ahmed Monib, Yousef Hawas, Abdullah Raizah, Turki Ahmed Alqahtani, Mahmoud El-Rosasy, Rashad G. Mohamad

**Affiliations:** 1 Faculty of Medicine, Cairo University, Cairo, Egypt; 2 Faculty of Medicine, Al-Azhar University, Cairo, Egypt; 3 Faculty of Medicine, Assiut University, Assiut, Egypt; 4 Faculty of Medicine, Elmergib University, Alkhums, Libya; 5 Faculty of Medicine, Tanta University, Tanta, Egypt; 6 Mansoura Manchester Program for Medical Education, Faculty of Medicine, Mansoura University, Mansoura, Egypt; 7 Faculty of Medicine, Benha University, Benha, Egypt; 8 Faculty Of Medicine, Jordan University of Science & Technology, Irbid, Jordan; 9 Department of Pharmacology and Toxicology, Faculty of Pharmacy, Tanta University, Tanta, Egypt; 10 Faculty of Medicine, Suez Canal University, Ismailia, Egypt; 11 Department of Orthopedics, Tanta University, Tanta, Gharbia Egypt; 12 Orthopedic Department, College of Medicine, King Khalid University, Abha, Saudi Arabia

**Keywords:** Carpal tunnel syndrome, Conventional open carpal tunnel release, Endoscopic carpal tunnel release, Ultrasound-guided carpal tunnel release (CTR-US), Double tunnel release

## Abstract

Carpal Tunnel Syndrome (CTS) is a common peripheral nerve entrapment disorder with a high global burden. Various surgical techniques have been developed to optimize symptom relief and minimize postoperative morbidity. This network meta-analysis (NMA) aimed to compare the relative efficacy and safety of different carpal tunnel release (CTR) procedures. We conducted a systematic review and NMA of randomized controlled trials (RCTs) comparing surgical CTR techniques. Outcomes included symptom severity, pain scores, functional status, patient-reported outcomes, operative time, and adverse events. Data were synthesized using the frequentist approach with the *netmeta* package in R. Confidence in network estimates was evaluated using the CINeMA framework. Thirty-two RCTs comprising 2,916 patients were included. One-port and two-port endoscopic CTR (ECTR) techniques demonstrated superior symptom relief compared to conventional open CTR (COCTR), particularly at three months (two-port ECTR: SMD = –4.47; 95% CI: –5.67 to –3.26). One-port ECTR consistently showed better functional outcomes across all timepoints, including improved grip (SMD = 1.37; 95% CI: 0.35 to 2.39) and pinch strength. Pain was significantly reduced with two-port ECTR at one and three months. Ultrasound-guided CTR (CTR-US) showed the highest patient satisfaction (OR = 6.89; 95% CI: 1.87 to 25.43) and a significantly shorter return-to-work duration. The double tunnels technique (DTT) had the lowest risk of adverse events (OR = 0.05; 95% CI: 0.01 to 0.42). Operational time and scar tenderness were comparable across all techniques. Heterogeneity was generally low to moderate across outcomes, and CINeMA assessments indicated moderate to low confidence in several comparisons. Endoscopic techniques, particularly one- and two-port ECTR, offer superior outcomes in symptom severity and functional recovery compared to COCTR. CTR-US appears to maximize patient satisfaction and early return to work, while DTT is associated with the fewest complications. These findings can guide individualized, evidence-based treatment decisions in CTS surgery.

**Supplementary Information:**

The online version contains supplementary material available at 10.1007/s10143-025-03587-x.

## Introduction

One of the most prevalent peripheral nerve entrapment syndromes is carpal tunnel syndrome (CTS) [1]. A recent meta-analysis found that the prevalence of CTS was 14.1% out of 53 million subjects [2]. Females aged 40–60 years are commonly affected with CTS, with a male-to-female ratio of 1:2–5. Also, the incidence rates in the US range from 0.5% to 1.0% [3]. Numbness, paresthesias, discomfort, thenar muscle atrophy, and functional deficiencies including pinch/grip weakness may be experienced by those who are affected [4]. The transverse carpal ligament (TCL) is thought to cause localized compression of the median nerve, which results in the clinical symptoms of CTS [4]. Carpal tunnel release (CTR), corticosteroid injection, and immobilization are appropriate alternatives for managing CTS [5].

From non-surgical to surgical therapy, there are currently various therapeutic approaches accessible for the diagnosis and treatment of CTS [6]. These approaches have been taken from different angles and with diverse methodologies as there are anatomical variations of the median nerve that may influence symptoms during the operation [7]. Resolving the median nerve's compression condition is the main goal of the surgical procedure [1, 3, 8]. The clinical presentation and physical examination serve as the basis for the diagnosis, which can be verified by electrophysiological testing, particularly electroneuromyography (ENMG), which measures sensory and motor delay as well as conduction abnormalities [4, 9–11]. Patients with mild CTS symptoms, non-steroid anti-inflammatory medications, vitamin B6, local steroid injections, or hand braces are the conservative treatment options, however, Patients with moderate to severe symptoms usually require surgical intervention [4, 12].

CTS surgery is one of the most common operations done. Around 350,000 CTR procedures are carried out annually in the US [13]. The standard method for decompressing the median nerve in cases of CTS is conventional open carpal tunnel release (COCTR) [14]. However, this procedure has several side effects, such as pillar pain, scar tenderness, cosmetic dissatisfaction, loss of grip and pinch strength, and missed work time [14–16]. In order to decompress the carpal tunnel with reduced postoperative scar discomfort and good skin cosmetic results, the limited-open carpal tunnel release (LOCTR) and double tunnels technique (DTT) has been found to be safe, dependable, and effective [14, 17, 18]. Also, endoscopic carpal tunnel release (ECTR) was proposed to lower the frequency of these issues [19]. Single-portal and two-portal endoscopic methods have been developed for the release of CTS [20]. Both may be more beneficial than COCTR because they can help patients get back to work sooner and reduce the frequency and intensity of pillar and scar tenderness [21]. Moreover, ultrasound guidance carpal tunnel release (CTR-US) has been shown to be similar to mini-open carpal tunnel release (mOCTR) in terms of efficacy, safety, and functional outcomes, though differences exist in specific recovery metrics and procedural characteristics [22].

Current research on CTR lacks direct comparisons between different techniques, and long-term safety data. In addition to the inconsistent reporting of results. CTR-US has been showing promising results but is mainly studied in small, non-randomized trials. Also, post-operative care has not been adequately assessed [59].

This NMA aims to comprehensively assess symptom severity, functional status, patient-reported outcomes, Operational time, and adverse events or complications related to the interventions in CTR surgery [23], between all CTR procedures; COCTR, LOCTR, mOCTR, DTT, one-port ECTR, two-port ECTR, and CTR-US, for treating CTS and supporting clinical decision.

## Methods

### Search strategy and data sources

We conducted a comprehensive search of electronic databases, including PubMed, Scopus, Embase, Cochrane Library, and Web of Science, from inception to March 2025. The search strategy included keywords and Medical Subject Headings (MeSH) terms related to carpal tunnel syndrome and interventions as follows: (Carpal Tunnel Syndrome OR Median Neuropathy OR Carpal tunnel) AND (Ultrasound guided OR mini-open OR traditional open OR open release OR open OR endoscopy OR endoscopic OR Ultrasonography OR surgery OR Surgical treatment OR Surgical intervention). This review's predetermined protocol is registered with PROSPERO under the CRD42024509242. The obtained records were aggregated and examined for duplicates using EndNote. Following"the Cochrane Handbook"recommendation [24], we performed this NMA and reported it using the NMA-preferred reporting item [25] (Electronic supplementary material (ESM) 1).

### Selection criteria

We incorporated studies that fulfilled the subsequent criteria:

(1) Population: All patients with either unilateral or bilateral Carpal Tunnel Syndrome or Median Neuropathy, at any severity level. However, as surgical intervention is generally reserved for moderate to severe CTS cases, the majority of included studies focused on these patients as the primary target population, not mild cases. (2) Interventions and comparators: Only include surgical interventions (e.g., COCTR, LOCTR, mOCTR, DTT, one-port ECTR, two-port ECTR, and CTR-US). Non-surgical interventions, such as physical therapy, exercises, splinting, or corticosteroid injections, were excluded to ensure comparability and homogeneity across surgical techniques and to maintain the transitivity of our analysis. (3) Study design: We limited the scope of our search to randomized controlled trials (RCTs) alone, to ensure high-quality evidence and comprehensive analysis. We excluded non-randomized studies, observational studies, case reports, case series, editorials, conference proceedings, abstracts or publications without full-text availability, non-English language studies, grey literature and studies not involving human subjects.

### Outcomes of interest included

(1) Symptom severity: pain score, scar tenderness, and Boston carpal tunnel questionnaire symptom severity scale (BCTQS). (2) Functional status: grip and pinch strength, two-point discrimination, distal Motor Latency (DML), and Boston carpal tunnel questionnaire functional status scale (BCTQF). (3) Patient-reported outcomes: satisfaction, return to work, and quality of life. (4) Operational time. (5) All adverse events reported in the included studies were extracted. These events varied in terminology and reporting style, so for consistency, we grouped them as reported by study authors without applying new classifications. Commonly reported events included scar tenderness, infection, recurrence of symptoms, and other complications related to wound healing or nerve symptoms. No additional categorization (e.g., intraoperative vs. postoperative) was applied due to inconsistent reporting across studies.

### Study selection and data extraction

Independent reviewers screened titles and abstracts of retrieved articles in duplicates to identify potentially relevant studies. The eligibility of the full-text articles was then evaluated following the predetermined inclusion criteria. Two reviewers independently extracted the data in duplicate using a standardized data extraction form, and a third reviewer validated the results. Extracted data included research characteristics (e.g., author, year of publication, study design), participant demographics, intervention specifics, outcome measures, and domains of Cochrane random risk of a bias assessment tool for RCTs. Any discrepancies between reviewers were first discussed to reach consensus. If disagreement persisted, a majority vote among the three reviewers was conducted. In cases where the vote was inconclusive or further clarification was needed; the senior author made the final decision.

### Quality assessment

The methodological quality of the included studies was assessed using appropriate tools such as the Cochrane Risk of Bias Tool for RCT (ROB-2) [26]. All five ROB-2 domains were assessed for each included study: (1) randomization process, (2) deviations from intended interventions, (3) missing outcome data, (4) measurement of the outcome, and (5) selection of the reported result. Each domain was rated independently by two reviewers. The overall risk of bias for each study was determined using the ROB-2 algorithm: if any domain was rated “high risk,” the study was classified as having high risk of bias; if one or more domains had “some concerns” but none were high risk, the study was rated as having “some concerns”; otherwise, it was rated low risk. Disagreements were resolved via discussion, and when necessary, by arbitration from the senior author.

### Data synthesis and statistical analysis

We utilized R statistical software (v4.3.2) and netmeta package (v2.8-2), to perform frequentist NMA. For continuous outcomes, we assembled the data as standardized mean difference (SMD), and for dichotomous outcomes, as odds ratio (OR) with a 95% confidence interval (CI) for each outcome. We investigated and measured significant heterogeneity with the Chi-squared (Q^2^) and I-squared (I^2^) tests, respectively. For each predetermined outcome, we created a forest plot and characterized the significant heterogeneity as a Breslow-Day Test I^2^ > 50% or P-value < 0.1. The substantial heterogeneity was resolved using sensitivity analysis to explore the sources of heterogeneity. A league table was created by comparing and organizing all the estimated values that our NMA was able to obtain, furthermore, we investigated the integrity of the network estimates by comparing the direct and indirect estimates using the netsplitting approach and assessed the transitivity of the network by comparing all the clinical and methodological variables that might modify the effect of the interventions. We applied the Confidence in Network Meta-Analysis (CINeMA) framework to assess the confidence in network estimates across six domains: within-study bias, reporting bias, indirectness, imprecision, heterogeneity, and incoherence. Judgments were based on ROB-2 assessments, width of confidence intervals, directness of evidence, heterogeneity (I^2^), and network inconsistency. A summary of these ratings is presented in the Supplementary Material (ESM 12, 13).

## Results

### Study selection and characteristics

**Fig. 1:**
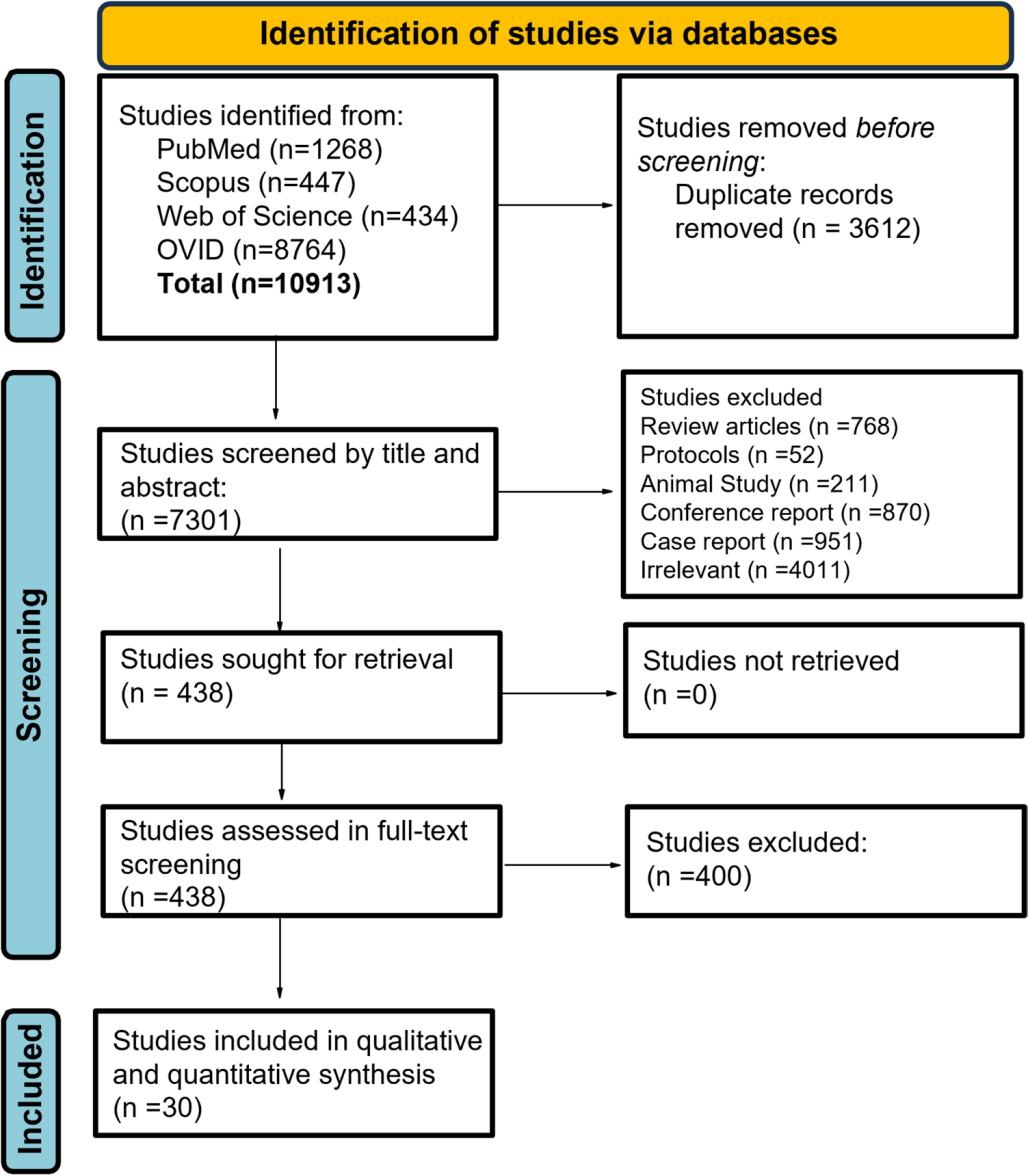
The search strategy flowchart

Our comprehensive search yielded 11,194 studies. After removing duplicates, a total of 7,375 articles were assessed for their eligibility based on their titles and abstracts. Following the removal of 6,863 articles that did not meet the eligibility criteria, the remaining 202 studies went through the full-text screening. Finally, 32 studies met our inclusion criteria [18, 21, 22, 27–53, 60, 61] (Fig. 1). The 32 studies involved 2,916 patients with a total of 3240 hands. The summary of included studies and baseline characteristics are shown in Tables 1 and 2 respectively.

**Table 1:** Summary of included studies

Study ID	Study arms	Country	Number of centers	Number of operated hands	Total of Participants	No. of participants/hands in each arm	Key findings	Follow up durations	Main inclusion criteria	Outcomes	Conclusion
Brown 1993 [27]	Agee two-portal endoscopic carpal tunnel release VS Standard volar incision conventional open carpal tunnel release under loupe magnification	USA	4	169	145	**two-port ECTR:** 76 **OCTR:** 75	Both methods provided high levels of symptom relief and patient satisfaction. Endoscopic group had less scar tenderness and faster return to work. Endoscopic group had a higher rate of complications, including nerve and vascular injuries.	3, 6, and 12 weeks	Patients with idiopathic CTS, failure, or refusal of non-operative treatment	Symptomatic relief and overall satisfaction of the patient after the procedure	Both OCTR and two-port ECTR achieved high levels of primary outcomes, but ECTR resulted in less scar tenderness and shorter return to work. However, ECTR had a higher rate of complications
Suppaphol 2012 [28]	Direct-vision limited open carpal tunnel release using a blunt tissue separator VS Standard volar incision conventional open carpal tunnel release	Thailand	2	30	28	**LOCTR:**15 **COCTR:** 15	No significant differences in symptom severity, functional scores, or two-point discrimination. Limited open group had better grip strength at 3 months and shorter wound length.	1, 3 months	Patients with clinically diagnosed with CTS and were confirmed by electrodiagnosis and had moderate to severe degrees that required operative treatment.	Levine’s symptom severity and functional score, grip, and pinch strength, and average two-point discrimination.	The LOCTR is effective and comparable to COCTR. The other advantages of this technique are better cosmesis and improvement in grip strength during the three-month postoperative period
Atroshi 2009 [29]	Two-portal ECTR using Smith & Nephew system (1 cm incisions) VS Standard open release with 4-cm longitudinal incision	Sweden	1	128	128	**two-port ECTR:** 63 **OCTR:** 65	ECTR resulted in less postoperative pain in the scar and palm, but without significant difference. No significant differences in return to work, symptom severity, or functional status.	3,6 weeks and 3,12 months	Patients with primary idiopathic CTS, age 25–60 years, currently employed, duration of symptoms of at least three months, inadequate response to six weeks'treatment with wrist splint, symptoms of classic or probable carpal tunnel syndrome according to the diagnostic criteria in the Katz hand diagram and nerve conduction test showing median neuropathy at the wrist (distal motor latency ≥ 4.5 milliseconds, wrist-digit sensory latency ≥ 3.5 milliseconds, or sensory conduction velocity at the carpal tunnel segment < 40 meters/second)	severity of postoperative pain experienced in the scar and proximal palm and the degree to which activity-related pain in the scar and palm or tenderness caused limitation of activity.	two-port ECTR has been associated with reduced postoperative discomfort than COCTR, but its cost-effectiveness is questionable due to the small amount of benefit and comparability with other outcomes.
Wong 2003 [30]	Modified Chow two-portal endoscopic release with retrograde hook knife VS Limited open release with specialized strippers and front-cutting knife (Indiana technique)	China	1	60	30	**two-port ECTR:** 30 hands **LOCTR:** 30 hands	No difference between the two groups at 1 year, but LOCTR had less scar tenderness and pillar pain the early postoperative period.	2, 4, 8,16 weeks, and at 6 and 12 months	Patients with bilateral idiopathic CTS	Wound and pillar pain grip and pinch strength and scar tenderness	Both strategies produced results that were comparable after a year of follow-up. At the second and fourth postoperative weeks, the scar in the LOCTR group was noticeably less painful. After LOCTR, the thenar and hypothenar (pillar) pain decreased as well.
Cellocco 2005 [31]	*Knifelight-assisted mini-open release vs. conventional open release*	Italy	1	100	86	**mOCTR:** 99 **LOCTR:** 123	mOCTR group had better early results in symptom relief and grip strength, but differences diminished by 30 months with lower recurrence rate in the mini-open group. mOCTR is safe and effective, with shorter recovery time.	1,3,6,12,30 months	Patients with CTS diagnosed via clinical examination and electrodiagnostic tests	BCTQ, (VAS) scale.	Both techniques were effective and safe for carpal tunnel release, but LOCTR had better results in terms of scar tenderness, grip strength, and patient satisfaction at 30 months follow-up.
Cresswell 2008 [32]	*Mini-open release using Indiana Tome vs. standard open release*	UK	1	N/A	200	**mOCTR:** 100 **OCTR:** 100	No significant differences in functional scores, pain, scar tenderness, or grip/pinch strength at 3 months between the two groups. Higher rate of complications and more recurrences at 7 years in the minimally invasive group.	1 and 2 weeks, 3 months, and 7 years	Adults between 18–59 years diagnosed with CTS	pain, scar tenderness, grip strength, pinch grip, and Levine–Katz questionnaire	mOCTR was slightly more effective than COCTR but much more expensive
Dumontier 1995 [33]	*Modified Chow two-portal ECTR (extrabursal) vs. palmar approach open release (3–4 cm)*	France	1	96	96	**two-port ECTR:** 56 **OCTR:** 40	No significant differences in pain relief, disappearance of paresthesiae, complications or time to return to work between the two groups. Endoscopic group showed better recovery of grip strength at 1 and 3 months.	2 weeks and 1, 3,6 months	Patients with CTS diagnosed via clinical examination and electrodiagnostic tests, with more than 1 month of follow-up	pain, disappearing of paraesthesia or time to return to work and grip strength	No statistically significant differences were found regarding pain, disappearance of paranesthesia or time to return to work. However, better recovery of grip strength was observed in the endoscopic group
Ferdinand 2002 [34]	*Two-portal ECTR with fallback to open vs. loupe-assisted open release*	UK	1	50	25	**one-port ECTR:** 25 hand **OCTR:** 25 hands	No significant difference in postoperative recovery between the two groups in terms of muscle strength, hand function, grip strength, dexterity, or sensation. No significant difference in complication rates.	6, 12, 26 and 52 weeks	Patients with bilateral idiopathic CTS, whose symptoms had persisted for more than three months despite the use of a night splint. Those with bilateral conduction delay at the carpal tunnel in the absence of any other abnormality.	Operating time, return of muscle strength and assessment of hand function, grip strength, manual dexterity or sensation, patient satisfaction with each wrist, complications.	In comparison with COCTR, one-port ECTR has a similar incidence of complications and a similar return of hand function but is a slightly slower technique to undertake.
Jugovac 2002 [35]	*Microscope-assisted open release with choledochoscope vs. Eversmann open release under loupe magnification*	Croatia	1	72	72	**LOCTR:** 36 **OCTR:** 36	Both techniques were equally effective and safe in terms of symptom relief. LOCTR had better recovery outcomes	3 months	Patients with a typical medical history of carpal tunnel syndrome hypoesthesia of 256 Hz for vibration sense, distal motor latency greater than 5 ms, and/or a sensory nerve conduction velocity less than 30 m/s.	Symptomatic relief after the procedure and Electrophysiological findings	LOCTR is as effective and safe as COCTR, but with better postoperative recovery and cosmetic results.
Kang 2012 [36]	*Agee single-port ECTR vs. mini-open release with subcutaneous tunnel (1.5 cm)*	Korea	1	104	52	**one-port ECTR:** 52 hands **mOCTR:** 50 hands	Both techniques showed similar improvement in function ((BCTQ) and DASH scores). More patients preferred the ECTR, primarily due to less scar-related or pillar pain. mOCTR was associated with more postoperative pain, while endoscopic had a higher incidence of transient symptom worsening​	3 months	Patients with electrodiagnostically confirmed, idiopathic, bilateral CTS	BCTQ and DASH scores	There were similar improvements in BCTQ and DASH scores after endoscopic and open techniques at 3 months postoperatively
Schwarm 2022 [37]	Retractor-endoscopic carpal tunnel release (Karl Storz) vs. standard open volar incision release (3–4 cm)	Germany	N/A	80	80 (SHOULD BE 40)	**ECTR:22** **OCTR:18**	In comparison to the OCTR, the ECTR produced better short-term results because of the greatly reduced length of surgery and postoperative pain, as well as improvements in neurophysiological data and the bishop rating score.	3 and 12 months	All patients had received conventional pharmacological treatments and physiotherapy before surgery and must be >18 with no history of any malignant disease, inflammatory disease, planned or current pregnancy, previous operation, or deformities on the affected side.	Modified Bishop rating system (BRS), incapacity to work, duration of postoperative pain, hypoesthesia, atrophy and subjective weakness.	The endoscopic procedure is safe as well as effective compared with conventional methods.
Chen 2021 [38]	Modified endoscopic CTR with 4 mm arthroscope vs. standard open release with S-shaped incision	China	N/A	94	94	**MECTR:48** **OCTR:46**	both MECTR and OCTR succeeded in reducing symptoms. Clinical effectiveness was the same, as indicated by the similar Kelly grading scores. However, MECTR offered several advantages, that included a quicker recovery from surgery, a shorter hospital stay, a quicker return to regular activities, and less scarring after surgery.	1, 2, 3, 13 months till 4 years	Typical symptoms (pain, hypesthesiaor paresthesia) on one hand, including the thumb, index or middle finger, for ≥2 weeks &the symptoms were unrelieved,recurrent and even aggravated after regular non‐surgical treatment for >3 months & Hamada Grade I or II; iv) fulfillment of the electrodiagnostic criteria or if they were not fulfilled, the presence of night pain that awoke the patients and a positive flick test were required.	Grip strength, Pinch strength and Two‐point discrimination	The use of one-port ECTR achieved higher patient satisfaction, a shorter operative time and hospitalization time. An earlier return to work time or resumption of a normal life, as well as less post‐operative scar pain compared with OCTR. Thus, these results suggested that one-port ECTR may be an effective method for the treatment of idiopathic CTS.
Cho 2015 [39]	Limited longitudinal open release (2 cm) vs. short transverse open release (1.5 cm wrist crease)	Korea	1	84	84	**OCTR:39** **LOCTR:40**	Over a two-year follow-up, both approaches showed comparable improvements in symptom severity. The two groups didn't differ significantly in terms of recurrence rates or post-surgical scar pain. The findings imply that there is no apparent difference between the two approaches'levels of effectiveness.	6 weeks & 3, 6 months, and 1 and 2 years	Clinical diagnosis of CTS confirmed by electrodiagnosis, moderate to severe degree requiring operative treatment	Brigham and Women’s Carpal Tunnel Questionnaire scores, scar discomfort, and subjective patient satisfaction.	Short wrist transverse open-release surgery showed similar early postoperative symptoms and subjective and functional outcomes to limited open release.
Larsen 2013 [40]	Linvatec one-portal ECTR vs. short mid-palm incision (3 cm) vs. classic long incision open release (7 cm)	Denmark	1	90	90	**ECTR:30** **OCTR (classic +short):60 (30+30)**	ECTR led to a faster return to work compared to OCTR, with no significant long-term differences. While ECTR showed early benefits in recovery, overall outcomes were similar across groups.	1, 2, 3, 6, 12, 24 weeks	Patients were older than 18 years, symptoms of paresthesia in two or more fingers innervated by the median nerve and of at least 3 month’s duration prior to inclusion, and electromyography and nerve conduction studies confirming carpal tunnel syndrome.	pain, paresthesia, grip strength, range of movement, pillar pain, and duration of sick leave	The endoscopic procedure was safe and had the benefit of faster rehabilitation and return to work. No major advantage to using a short incision could be found. No significant difference in pain, paresthesia, range of motion, pillar pain, and grip strength could be found at 24 weeks of follow-up.
Macdermid 2003 [41]	Chow two-portal ECTR vs. standard open long-incision release	Canada	1	123	123	**ECTR:91** **OCTR:32**	The study found that neither approach had any appreciable long-term benefits.Long-term satisfaction was slightly lower in the endoscopic group.both procedures led to similar improvements in symptoms and had no significant differences in complication rates.	1, 6, 12 weeks	patients were deemed appropriate for surgery (i.e., had a poor response to 6 months of conservative management, splinting and change in activity) and had electrodiagnostic testing that confirmed the presence of carpal tunnel syndrome	Symptom Severity Scores and grip strength, physical health, key pinch strength, and tripod pinch strength	there isn't a significant difference in benefit between the two methods, with the endoscopic method showing lower long-term satisfaction due to re-operation rates
Eberlin 2022 [22]	CTR-US using UltraGuideCTR® device VS mini-open using subcutaneous tunnel	USA	11	122	149	CTR-US**:94** mOCTR**:28**	no major differences in outcomes. CTR-US showing a slight advantage in reduced wound sensitivity and pain. The study concluded that the choice between techniques should be guided by shared decision-making between patient and physician.	3 Months	Patients with a clinical diagnosis of idiopathic CTS, CTS-6 score ≥ 12, median nerve cross-sectional area ≥10 mm^2^ in the proximal carpal tunnel region, absence of CTS in the contralateral hand that interfered with normal daily activities or work, and failure of nonsurgical treatment.	Time to return to normal daily activities, time to return to work in any capacity among employed patients, BCTQ, Symptom Severity Scale and Functional Status Scale, Numeric Pain Scale, EuroQoL-5 Dimension 5-Level, and device- or procedure-related adverse events (AEs).	The efficacy and safety of CTR-US were comparable to mOCTR despite less previous surgical experience with CTR-US, both treatment groups reported rapid return to work and activity, statistically significant and clinically important improvements in symptoms, function, and health-related quality of life, with low complication rates, through 3 months. Overall, the choice of CTR technique should be determined by shared decision-making between the patient and physician.
Michelotti 2018 [42]	*Agee one-portal ECTR vs. standard open release (3 cm palmar incision)*	USA	1	60	30	**ECTR:30** **OCTR:30**	The results showed no significant differences.most patients (80%) subjectively preferred the endoscopic technique, citing less pain and faster recovery as reasons, even though pain scores were not significantly different.	24 weeks	patients aged 18 to 75 years with clinical and electrodiagnostic testing confirmation of bilateral carpal tunnel syndrome.	CTS functional status score (and the carpal tunnel syndrome symptom severity score	Both techniques are well tolerated with no differences in outcomes. With the added cost and equipment associated with one-port ECTR, and no added benefit, the usefulness of one-port ECTR is questionable.
Oh 2017 [43]	*Agee ECTR vs. mini-open release with nasal speculum (1.5 cm incision)*	South Korea	1	67	67	**ECRT:35** **MI-OCRT:32**	The study found that in patients with carpal tunnel syndrome, both methods are equally successful in reducing symptoms and reversing nerve damage.	24 weeks	Patients who are 20 years or older, with idiopathic CTS that was confirmed by electrodiagnostic tests, and who were scheduled for carpal tunnel release.	BCTQ	Both mOCTR and one-port ECTR significantly reversed the pathological changes in the median nerve morphology of patients with CTS, with no significant differences between techniques.
Uchiyama 2002 [44]	Double Tunnels technique (0.6 cm incision) vs. standard open interthenar release (3 cm)	Japan	1	66	66	**ECTR:33** **OCTR:30**	both resulted in similar improvements in nerve conduction over a 12-month period. the study suggests that OCTR may achieve slightly faster initial improvement, although this was not statistically significant.	12 months	patients who complained of intermittent or constant numbness or paresthesia over the distribution of the median nerve in the hand, positive Phalen’s provocation test and sensory disturbances in the distribution of the median nerve area, abnormal sensory conduction velocity (SCV), and motor distal latency (MDL) MDL ranged from 5 to 10 ms.	improvement of motor distal latency (MDL), sensory nerve conduction velocity (SCV) of the median nerve, and the amplitudes of compound muscle action potential (CMAP) and sensory nerve action potential (SNAP)	There is a risk of nerve damage in patients undergoing two-port ECTR. Although statistical analysis suggests that nerve conduction improves by about the same degree 12 months after ECTR or OCTR.
Vanni 2015 [18]	Open release with subneural reconstruction vs. standard open release	Italy	1	220	220	**DTT:110** OCTR**:110**	Faster healing from wrist discomfort, night pain, numbness, stiffness, and weakness was demonstrated by the results using DTT. Additionally, it reported better aesthetic results and better pain scores on the BCTSQ and VAS. Additionally, those who received DTT experienced fewer complications and went back to work sooner.	12 months	Adult patients (18 years or older) with a clinical diagnosis of CTS,	the functional Boston Carpal Tunnel Syndrome Questionnaire (BCTSQ) scores and visual analog scale (VAS) scores for pain.	The DTT is a safe and effective approach for the treatment of CTS. This technique entails faster recovery times, better aesthetic outcomes, and lower risks of complications.
Zhang 2015 [45]	Chow two-portal ECTR vs. double-incision under loupe vs. standard open release	China	3	213	213	**ECTR:53** OCTR**:160 (92+68)**	all three techniques led to similar improvements in symptoms the TCL reconstruction group showed superior outcomes endoscopic release resulted in less scar pain and better aesthetic outcomes	24 months	Patients had symptoms of CTS lasting at least three months or an inadequate response to conservative treatment after at least six weeks.	the severity of symptoms, lateral grip strength, pinch grip strength and Michigan Hand outcome scores	No significant difference between the groups in terms of severity of symptoms or lateral grip strength.
Zhang 2016 [46]	CTR via longitudinal incision with LRTI vs. CTR via separate longitudinal incision with LRTI	China	1	207	207	**ECTR:69** OCTR**:138 (73+65)**		2 years	Patients with a confirmed diagnosis of CTS based on Evidence for Surgical Treatment issued by the British Society for Surgery of the Hand (2003) and symptoms of CTS had lasted 2–11 months or inadequate responses to the conservative treatments for at least 3 months; and moderate to very severe symptoms.	severity of symptoms, Functional status using the Levine-Katz Questionnaire, and cylindrical, lateral, and pinch grip strength	Carpal tunnel release via double small incisions is a mini-invasive and less technically challenging procedure with good nerve visualization, resulting in a good appearance of scars.
Esteban‐Feliu 2022 [47]	CTR via FCR tendon incision during LRTI vs. CTR via separate longitudinal palmar incision post-trapeziectomy	Spain	1	40	40	**single incision:20** **double incision:20**	Patients in both groups experienced similar reductions in BCTQ scores, QuickDASH scores, and VAS pain ratingsHowever, the single-incision approach had the advantage of significantly shorter surgery time and none of the patients experienced pillar pain and it achieved slightly higher strength	at 2 and 6 weeks, and 3, 6 and 12 months	patients scheduled for surgical treatment of both primary basal joint osteoarthritis and CTS in the ipsilateral extremity.	the Boston Carpal Tunnel Questionnaire, QuickDASH, and a 10-point visual analog scale pain-severity rating	Our results supported the potential use of COCTR as an alternative to DTT when combining CTR and trapeziectomy.
Saw 2003 [48]	*Single-portal Agee ECTR (MicroAire) vs. standard open release (2 cm palmar incision)*	UK	3	150	150	ECTR **:74** OCTR**:76**	Both procedures showed similar improvements However, employed patients in the ECTR group returned to work an average of eight days sooner than those in the OCTR groupThe study concluded that ECTR is a cost-effective option for employed patients due to the faster return to work, but may not be as beneficial for the general population.	at 1, 3, 6, and 12 weeks	patients with a diagnosis of CTS. The diagnosis of carpal tunnel syndrome was made on clinical grounds. Nerve conduction tests were performed only when there was clinical doubt	Symptom Severity and Functional Status Scales questionnaires, A visual analog scoring system, Grip strength, Days off work	We conclude that two-port ECTR has the advantage of a quicker return to work for those in employment and provides an economic benefit.
Rojo-manaute 2016 [49]	Percutaneous ultrasound-guided release (≤1 mm) vs. mini-open release (2 cm curved incision)	Spain	1	92	92	**Mini-OCTR:46** **Ultra-MIS:46**	The results showed that the ultra–minimally invasive approach led to significantly better functional outcomes and patients experienced faster recovery, with a quicker return to normal daily activities They also discontinued analgesics significantly sooner Postoperative pain was also lower	1, 3, and 6 weeks and 3, 6, and 12 months	Eligible participants had clinical signs30 of primary carpal tunnel syndrome and positive electrodiagnostic test results.	Quick DASH questionnaire, Grip strength and time for discontinuation of oral analgesics, complete wrist flexion-extension, relief of paresthesia, and return to normal daily activities	CTR-US provides earlier functional return and less postoperative morbidity with the same neurologic recovery as mOCTR for patients with symptomatic primary carpal tunnel syndrome.
Rab 2006 [50]	Two-portal ECTR (3.5 cm) vs. standard open release (5 cm)	Austria	1	20	10	ECTR **:10** OCTR **:10**	The study found no statistically significant differences between them Both techniques led to significant improvements in pain severity, symptom severity, and nerve function parameters after one year, with grip and pinch strength either improving or remaining unchanged.	at 2, 4, 6, and 12 weeks and after 6 and 12 months	patients with bilateral idiopathic CTS diagnosed by positive history and examination of the patient (night pain, tinel’s sign at the wrist, median nerve sensory disturbances) and positive electrophysiological studies and sensory antidromic conduction velocity on each side	VAS, Levine-Score, Grip strength, pinch grip strength	The two-port ECTR revealed no distinct advantages over the COCTR not only in the late but also in the early postoperative follow-up period when performing intra-individual comparison.
Hamed 2009 [51]	Two-portal ECTR (3.5 cm) vs. standard open release (5 cm)	United Kingdom.	1	40	40	**single incision OR:21** **double incision OR:19**	Comparing the double-incision approach to the single-incision technique, the results indicated that the former greatly decreased scar sensitivity and pillar pain. But there was no discernible change in the recovery of grip strength.y	10 to 12 d, 6 weeks, and 3 and 6 months	patients in whom CTS was diagnosed both on clinical and neurophysiological backgrounds.	pillar pain, scar sensitivity, and recovery of grip strength	the DTT offered over the COCTR, including a reduction in both pillar pain and scar sensitivity, which often impede patients’ daily activities.
Palmer 1993 [21]	Two-portal ECTR (3.5 cm) vs. standard open release (5 cm)	U.S. A	1	211	163	one-port ECTR**:90** two-port ECTR:72 OCTR**:49**	There was no discernible difference between the three approaches in terms of how well paresthesias or nocturnal discomfort resolved. Although there are still worries about possible consequences and a steep learning curve for endoscopic treatments, the study found that endoscopic techniques are superior to open release in terms of recovery time and pain reduction..	at 2, 4, and 6 weeks, and 3 and 6 months	All patients had one or more symptoms typical of CTS (numbness, tingling, weakness, and pain at night or with activity). All patients had abnormal nerve conduction study results. All patients had failed prior treatment with splinting and nonsteroidal anti-inflammatory medication for at least 2 months before their release.	symptoms of paresthesia, night pain, and thumb weakness, pain with activities of daily living on a 10-cm visual analog scale, Weber two-point, grip strength, pinch strength, Scar tenderness, return to work (days) after procedure	Proponents of one-port ECTR claimless pain and scar tenderness, quicker recovery of strength, and earlier return to work and daily activities over COCTR.
Sennwald 1995 [52]	Agee single-portal ECTR vs. open release with retinaculum reconstruction (Sennwald technique)	Switzerland	1	47	47	ECTR **:25** OCTR **:22**	The endoscopic method led to a quicker restoration to normal hand function and a noticeably improved recovery of grip strength. Although the study found that endoscopic release reduces morbidity and speeds up recovery, it also highlighted the need for improvements to improve its safety and diagnostic potential.	4, 8, and 12 weeks.	the only criterion of choice was the surgical procedure	pain relief, grip, key-pinch, strength, and ability to return to work.	The findings were strongly in favor of one-port ECTR. However, this technique does not allow any analysis of the pathology or structure to be treated.
Fuente 2021 [53]	Agee single-portal ECTR vs. open release with retinaculum reconstruction (Sennwald technique)	Spain	1	89	89	**USCTR :47** OCTR **:42**	At three months, the group that received ultrasound guidance had noticeably improved hand functionality and reduced pain. Both groups took about the same amount of days off from work. Even so, the group that received ultrasound guidance experienced slightly more difficulties. Although the therapeutic significance of these changes is still restricted, the study found that both approaches are beneficial, with the ultrasound-guided strategy providing advantages in functionality and pain reduction.	at 3, 6, and 12 months	Inclusion criteria were: (a) age 20 to 65 years; (b) clinical signs of carpal tunnel syndrome; (c) diagnosis of primary carpal tunnel syndrome confirmed by electroneurography (ENG).	Boston Carpal Tunnel Syndrome Questionnaire (BCTQ-S	The findings reveal similar symptom relief benefits following OCTR or CTR-US. However, CTR-US had better hand functional status and less pain.
Nakamichi 1997 [60]	*Ultrasound-guided release via basket punch (Safe-line) vs. curved open release (5 mm ulnar to thenar crease)*	Japan	1	103	103	**UCTR: 50/OCTR: 53**	UCTR had better early pain, tenderness, cosmetic scar, grip/pinch strength; no differences in other outcomes	3, 6, 13, 26, 52, 104 weeks	Female homemakers with idiopathic CTS, refractory to conservative treatment >3 months	Numbness, paresthesia, 2PD, monofilament, APB strength, grip, pinch, scar, electrophysiology	UCTR is safe/effective, better early recovery, scar, equal neuro outcomes vs OCTR
Alvarez 2024 [61]	Z-plasty vs Conventional surgery (complete TCL opening)	Spain	1	109 (assumed)	109	**Z-plasty: 55/Conventional: 54**	Z-plasty had lower pillar pain at 3 weeks and better pinch strength recovery at 6 months	3 weeks, 6 months	Adults with moderate–severe CTS, failed conservative treatment	Pillar pain, grip/pinch strength, BCTQ clinical and functional scores	Z-plasty is a valid alternative, improves pillar pain and strength outcomes vs conventional

**Table 2:** Baseline characteristics of participants in the included studies

Study ID	Study Arms	Age (Mean ± SD)	Male patients *N* (%)	Body Weight (Mean ± SD)	Patients with Bilateral CTS *N* (%)	Dominant Arm Involved *N* (%)	No. of Employed Patients *N* (%)
Brown 1993 [27]	Intervention	Not Reported.	31 (40.7%)	Not Reported.	8 (10.5%)	52 (68.4%)	41 (54%)
	Control	Not Reported.	23 (3.7%)	Not Reported.	10 (13.3%)	52 (69.3%)	40 (53.3%)
Suppaphol 2012 [28]	Intervention	53.33	1 (6.7%)	Not Reported.	1 (3.5%)	Not Reported.	Not Reported.
	Control	53.13	6 (13.4%)	Not Reported.	1 (3.5%)	Not Reported.	Not Reported.
Atroshi 2009 [29]	Intervention	44	19 (30%)	27.5 **±** 4.5	Not Reported.	48 (76)	1
	Control	44	13 (20%)	26.7 **±** 4.4	Not Reported.	54 (83)	1
Wong 2003 [30]	Intervention	Not Reported.	2 (6.67%)	Not Reported.	30 (100%)	Not Reported.	0.4
	Control	Not Reported.	2 (6.67%)	Not Reported.	30 (100%)	Not Reported.	0.4
Cellocco 2005 [31]	Intervention	Not Reported.	19 (23%)	Not Reported.	Not Reported.	Not Reported.	Not Reported.
	Control	Not Reported.	31 (30%)	Not Reported.	Not Reported.	Not Reported.	Not Reported.
Cresswell 2008 [32]	Intervention	Not Reported.	Not Reported.	Not Reported.	Not Reported.	Not Reported.	Not Reported.
	Control	Not Reported.	Not Reported.	Not Reported.	Not Reported.	Not Reported.	Not Reported.
Dumontier 1995 [33]	Intervention	53.4 ± 14.49	7 (14.3%)	Not Reported.	Not Reported.	Not Reported.	21 (58%)
	Control	50.7 ± 13.98	4 (11.1%)	Not Reported.	Not Reported.	Not Reported.	24 (49%)
Ferdinand 2002 [34]	Intervention	54.88 (12.8)	5 (20%)	Not Reported.	25 (100%)	Not Reported.	17 (68%)
	Control	54.88 (12.8)	Not Reported.	Not Reported.	25 (100%)	Not Reported.	17 (68%)
Jugovac 2002 [35]	Intervention	54.2 ± 8.9	5 (13.89%)	Not Reported.	Not Reported.	Not Reported.	36 (100%)
	Control	52.5 ± 9.7	13 (36.11%)	Not Reported.	Not Reported.	Not Reported.	36 (100%)
Kang 2012 [36]	Intervention	55 ± 10	4 (7.7%)	Not Reported.	52	25 (48.1%)	14 (26.9%)
	Control	55 ± 10	4 (7.7%)	Not Reported.	52	27 (51.9%)	14 (26.9%)
Schwarm 2022 [37]	Intervention	60.33 ± 22.19	5 (22.7%)	Not Reported.	Not Reported.	12 (54.5%)	Not Reported.
	Control	55.7 ± 31.62	2 (11.1%)	Not Reported.	Not Reported.	11 (61.1%)	Not Reported.
Chen 2021 [38]	Intervention	50.8 ± 7.97	18 (37.5%)	Not Reported.	Not Reported.	Not Reported.	Not Reported.
	Control	48.0 ± 5.33	16 (34.78%)	Not Reported.	Not Reported.	Not Reported.	Not Reported.
Cho 2015 [39]	Intervention	53 ± 6.96	3 (7.5%)	Not Reported.	Not Reported.	21 (53%)	17 (42.5%)
	Control	55 ± 5.82	3 (7.7%)	Not Reported.	Not Reported.	20 (51%)	16 (39%)
Larsen 2013 [40]	Intervention	54 ± 11.5	8 (26.6%)	Not Reported.	Not Reported.	18 (60%)	16 (53.3%)
	Control	54 ± 10.7	12 (40%)	Not Reported.	Not Reported.	20 (66.7%)	17 (56.6%)
Macdermid 2003 [41]	Intervention	45 ± 15	29 (32%)	Not Reported.	Not Reported.	Not Reported.	21 (23%)
	Control	53 ± 16	10 (32%)	Not Reported.	Not Reported.	Not Reported.	8 (25%)
Eberlin 2022 [22]	Intervention	56.4 ± 14.6	39 (38.6%)	31.1 ± 7.5	28 (29.8%)	15 (58.5%)	56 (59.6%)
	Control	57.9 ± 13.5	16 (33.3%)	31.5 ± 7.9	13 (46.4%)	17 (60.7%)	19 (67.9%)
Michelotti 2018 [42]	Intervention	54	5 (17%)	Not Reported.	30 (100%)	13 (43%)	Not Reported.
	Control	54	5 (17%)	Not Reported.	30 (100%)	17 (57%)	Not Reported.
Oh 2017 [43]	Intervention	51.8 ± 10.4	5 (16%)	Not Reported.	28 (88%)	Not Reported.	6 (19%)
	Control	52.9 ± 10.6	5 (14%)	Not Reported.	25 (71%)	Not Reported.	8 (23%)
Uchiyama 2002 [44]	Intervention	57.1 ± 8.89	1 (3%)	Not Reported.	Not Reported.	Not Reported.	Not Reported.
	Control	56 ± 7.84	1 (3%)	Not Reported.	Not Reported.	Not Reported.	Not Reported.
Vanni 2015 [18]	Intervention	55.8	29 (26.36%)	Not Reported.	9 (8%)	93 (85%)	Not Reported.
	Control	54.9	28.87 (26.24%)	Not Reported.	7 (6%)	93 (85%)	Not Reported.
Zhang 2015 [45]	Intervention	44 ± 6.84	16 (30%)	Not Reported.	0	32 (60%)	23 (34%)
	Control	47 ± 7.69	33 (35.8%)	Not Reported.	0	55 (59.78%)	44 (48%)
Zhang 2016 [46]	Intervention	48 ± 6.97	25 (36.2%)	Not Reported.	0	Not Reported.	Not Reported.
	Control	45 ± 6.4	22 (33.8%)	Not Reported.	0	Not Reported.	Not Reported.
Esteban‐Feliu 2022 [47]	Intervention	61.5 ± 6.877	4 (20%)	Not Reported.	Not Reported.	9 (45%)	Not Reported.
	Control	58.45 ± 6.219	3 (15%)	Not Reported.	Not Reported.	12 (60%)	Not Reported.
Nakamichi 1997 [60]	Intervention	58 year (45–81)	0	Not Reported	Excluded	Not Reported	0 (All homemakers)
	Control	58 year (45–81)	0	Not Reported	Excluded	Not Reported	0 (All homemakers)
Alvarez 2024 [61]	Intervention	55.5 ± 10.8	10 (18.2%)	Not reported	Not reported	Right: 52.7%	Not reported
	Control	56.7 ± 12.7	19 (35.2%)	Not reported	Not reported	59.3%	Not reported

### Risk of bias of included studies

**Fig. 2:**
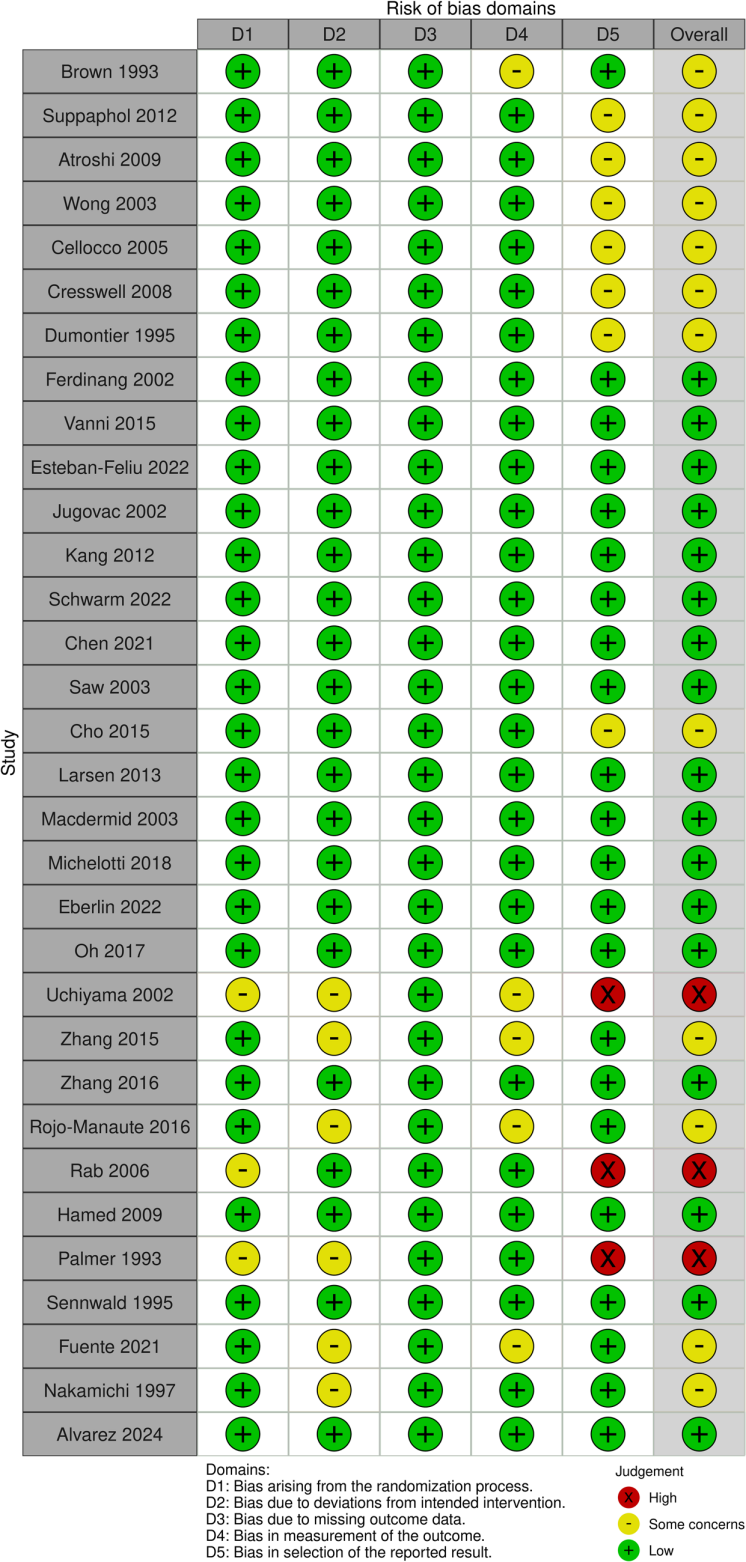
Quality assessment of the included studies according to the ROB-2 tool

The risk of bias was assessed using the ROB-2 tool for 32 included studies. 17 studies exhibited an overall low risk of bias. Also, 12 studies were deemed to raise concerns regarding bias in the selection of the reported outcome in seven studies, bias in measurement outcome in five studies, and bias due to missing outcomes in three studies. Moreover, three studies exhibited a high risk of bias. The detailed risk of bias summary and graph are in (Fig. 2).

### Results of symptoms severity

#### BCTQS

The analysis of symptom severity according to BCTQS demonstrated that, at one month, mOCTR and one-port ECTR showed statistically significant mean differences (SMD = −0.35, 95% CI: −0.62 to −0.08; *p* = 0.0101) (SMD = −1.42, 95% CI: −1.99 to −0.86; *p* < 0.0001), respectively, compared to COCTR, suggesting a notable beneficial effect associated with those procedures. While two-port ECTR displayed a more pronounced mean difference, it did not reach the conventional statistical threshold (SMD = −0.31, 95% CI: −0.65 to 0.03; *p* = 0.0740), indicating a potential reduction in symptom severity.

At three months, one-port ECTR also exhibited a statistically significant reduction in symptom severity while mOCTR did not (SMD = −1.30, 95% CI: −2.25 to −0.35; *p* = 0.0072) (SMD = −0.84, 95% CI: −1.73 to −0.06; *p* = 0.0678). Two-port ECTR demonstrated an exceptionally large and highly significant effect (SMD = −4.47, 95% CI: −5.67 to −3.26; *p* < 0.0001), suggesting a profound reduction in symptom severity. In contrast, CTR-US did not have a significant effect with a (SMD = −0.36, 95% CI: −1.14 to 0.43; *p* = 0.37).

At six months, all the studied procedures demonstrated similar symptom severity compared to COCTR except one-port ECTR which was the only significant intervention (SMD = −1.01, 95% CI: −1.98 to −0.05; *p* = 0.0394).

**Fig. 3:**
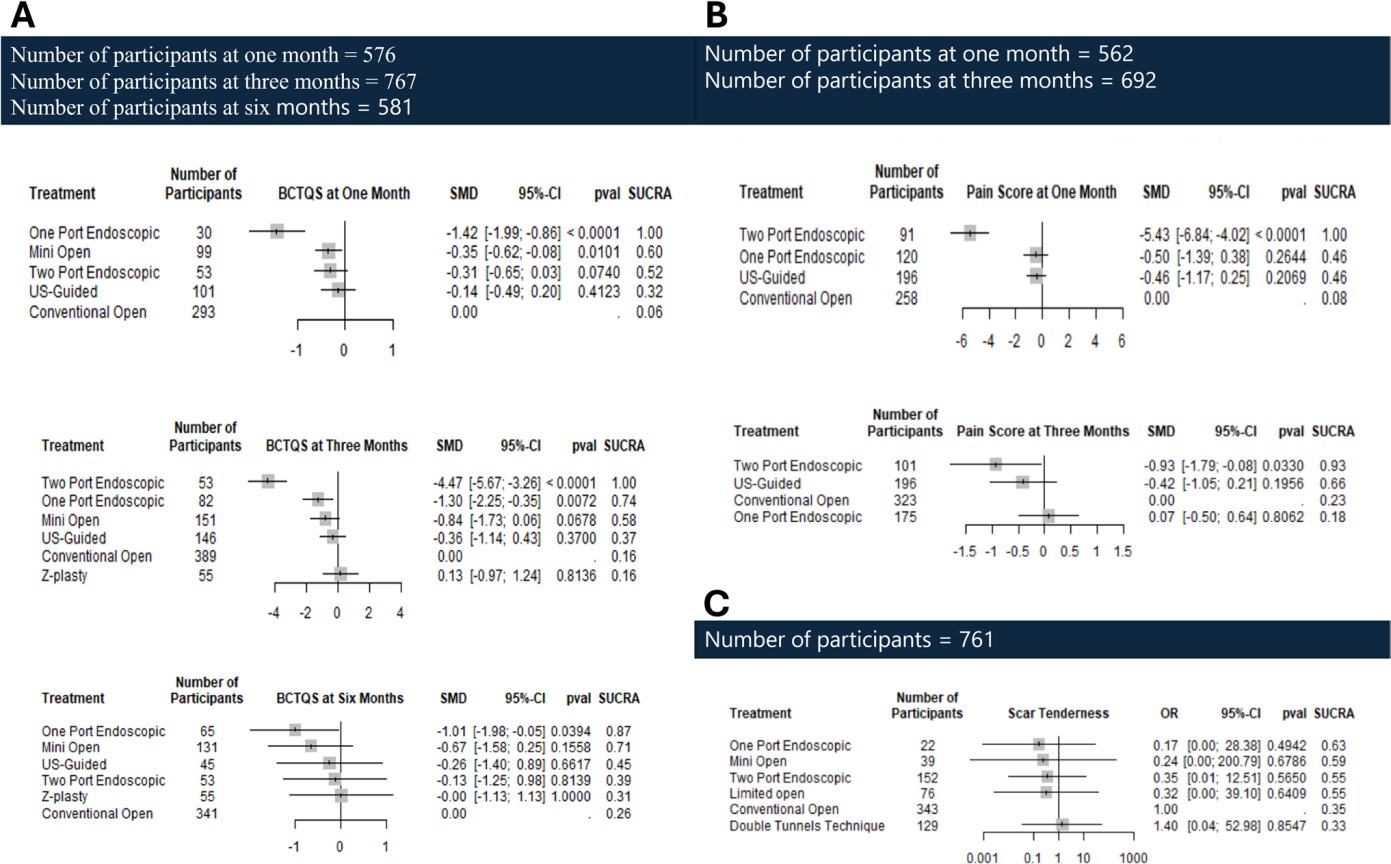
Forest plots of Symptoms Severity represented with mean difference (MD), confidence interval (CI), and standard deviation (SD) at; one, three, and six months for (**a**) BCTQFS, and (**b**) one, three months for pain (Vas-score); while forest plot of (**c**) Scar Tenderness represented with odds ratio

Heterogeneity analyses across all time points indicated minimal variability in treatment effects (I^2^= 0%, *P* > 0.05), further supporting the overall consistency of the findings (Fig. 3a).

##### Pain score

The analysis of pain score at one month revealed that two-port ECTR displayed the most pronounced decrease in pain score (SMD = −5.43, 95% CI: −6.84 to −4.02; *p* < 0.0001), while one-port ECTR and CTR-US did not show a statistically significant decrease in pain score, indicating no statistically significant difference compared to COCTR.

At three months, two-port ECTR continues to appear as the most effective treatment in decreasing pain score (SMD = −0.93, 95% CI: −1.79 to −0.08; *p* = 0.0330), while one-port ECTR and CTR-US exhibited similar pain scores compared to COCTR. Heterogeneity analyses indicated no observed variability in treatment effects (I^2^ = 0%, *p* = 0.411), further supporting the overall consistency of the findings. (Fig. 3b).

##### Scar tenderness

The analysis of scar tenderness across different procedures and COCTR revealed that DTT, LOCTR, mOCTR, one-port ECTR, and two-port ECTR exhibited comparable treatment effects to COCTR. Heterogeneity analyses indicated a significant level of variability in treatment effects (I^2^ = 79.6%, *p* = 0.0075), indicating significant variation in treatment effects both within and between the study designs (Fig. 3c).

NMA league plots for Symptoms severity at three months in (Fig. 5a), while at one and six months for BCTQS and only at one month for Pain (Vas-score) in (ESM. 2).

### Results of functional status

#### BCTQF

**Fig. 4:**
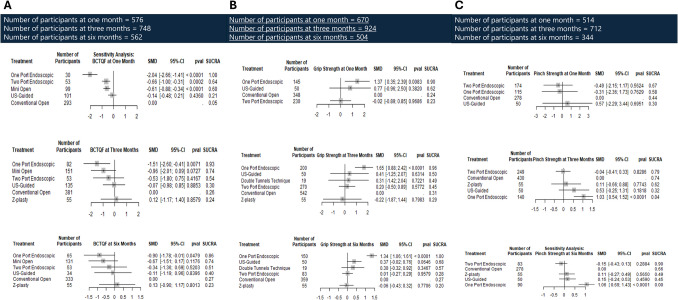
Forest plots of Functional Status represented with mean difference (MD), confidence interval (CI), and standard deviation (SD) at one, three, and six months for; **a** BCTQF, **b** Grip Strength, and (**c**) Pinch Strength

The analysis of the functional status according to BCTQF demonstrated that, compared to COCTR, one-port ECTR displayed the most pronounced decrease in BCTQF scale at all measured time points, while mOCTR and one-port ECTR also showed significantly lower scores at one month, indicating better functional status associated with these procedures. In contrast, CTR-US did not show a statistically significant difference compared to COCTR (SMD: −0.14; 95% CI: −0.48 to 0.21; *p* = 0.44). Heterogeneity analyses indicated minimal variability in treatment effects (I^2^ = 0%, *p* > 0.05), further supporting the overall consistency of the findings (Fig. 4a).

##### Grip strength

The analysis comparing grip strength revealed that one-port ECTR exhibited significantly higher grip strength across all measured time points compared to COCTR (SMD = 1.37, 95% CI: 0.35 to 2.39; *p* = 0.0083). In contrast, the DTT and two-port ECTR displayed similar grip strength across all time points compared to Conventional open release. Heterogeneity analyses indicated a high level of variability in treatment effects (I^2^ = 94.1%, *p* < 0.0001), indicating significant variation in treatment effects both within and between the study designs (Fig. 4b).

##### Pinch strength

The analysis of pinch strength at one month revealed that one-port ECTR and two-port ECTR did not show statistically significant differences compared to COCTR, while at three and six months, one-port ECTR exhibited significantly stronger pinch strength compared to COCTR (SMD = 1.03, 95% CI: 0.54 to 1.52; *p* < 0.0001), (SMD = 1.06, 95% CI: 0.69 to 1.43; *p* < 0.0001) respectively. In contrast, two-port ECTR did not show a significant difference compared to COCTR. Heterogeneity analyses indicated a moderate level of variability in treatment effects (I^2^ = 79.5%, *p* < 0.0001), indicating significant variation in treatment effects both within and between the study designs (Fig. 4c).

##### Two-point discrimination

The analysis of two-point discrimination at three months revealed that LOCTR exhibited a significant difference (SMD = −5.66, 95% CI: −7.26 to −4.06; *p* < 0.0001) compared to COCTR. one-port ECTR and two-port ECTR, on the other hand, did not show significant differences compared to COCTR (SMD = 0.04, 95% CI: −0.33 to 0.42; *p* = 0.8289) and (SMD = 0.07, 95% CI: −0.13 to 0.27; *p* = 0.5107), respectively. Heterogeneity analysis revealed minimal variability in treatment effects across the studies (I^2^ = 0%, *p* = 0.619), suggesting homogeneity in treatment effects within the individual study designs and overall (ESM. 3).

#### DML

The analysis of distal motor latency at three months revealed that one-port ECTR exhibited a significant mean difference (SMD = −0.63, 95% CI: −1.20 to −0.06; *p* = 0.0291). However, two-port ECTR and CTR-US demonstrated comparable scores of distal motor latency to COCTR (SMD = 0.14 CI: −0.36 to 0.63; *p* = 0.5877) and (SMD = 0.02, 95% CI: −1.27 to 0.73; *p* = 0.9005), respectively (ESM. 4).

**Fig. 5:**
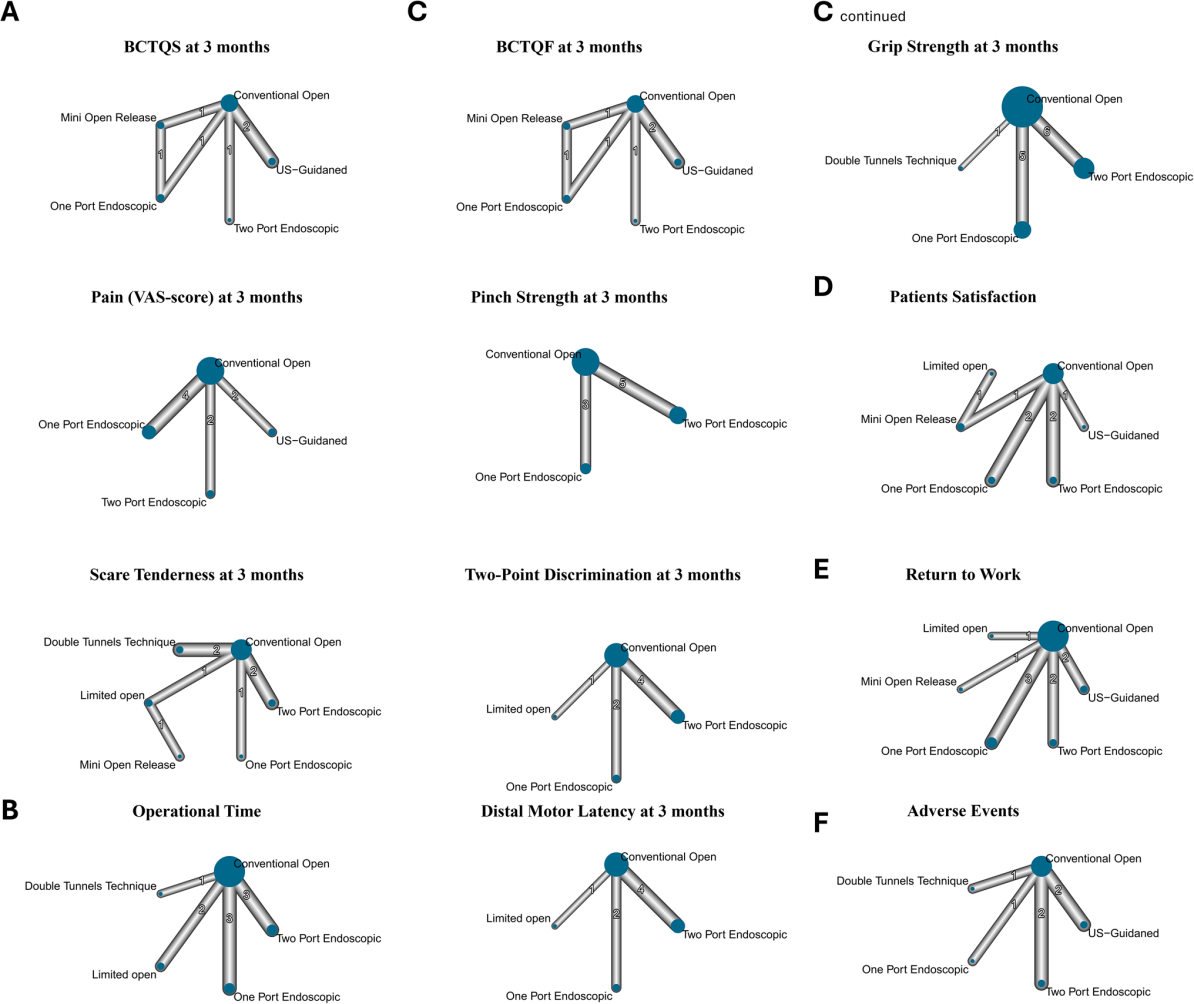
Network plots representing evidence directly are used in NMA. Nodes represent the procedures and edges represent the direct comparisons. Node size and line thickness are proportional to the number of papers providing direct evidence. **a** Symptoms Severity at 3 months, **b** Operational Time, **c** Functional Status at 3 months, **d** Patients Satisfaction, **e** Return to work**,** and (**f**) Adverse Effects

NMA league plots for Functional Status at three months in (Fig. 5c), while at one and six months for Grip Strength and Pinch Strength in (ESM. 5).

### Results of patient-reported outcomes

#### Patient satisfaction

The analysis of patients’ satisfaction across different treatments and COCTR revealed that CTR-US demonstrated the most significant odds ratio (OR) of 6.89, 95% CI: 1.87 to 25.43; *p* = 0.004), indicating a substantial increase in the odds of patient satisfaction compared to the COCTR. Heterogeneity analyses indicated a moderate level of variability in treatment effects (I^2^ = 33.8%, *p* = 0.221), indicating no significant variation in treatment effects either within or between the study designs (ESM. 6).

#### Return to work

The analysis of time required to return to work across different treatments and COCTR revealed that mOCTR, CTR-US and one-port ECTR had a significantly quicker return to work (SMD = −6.43, 95% CI: −10.61 to −2.26; *p* = 0.0025), (SMD = −3.99, 95% CI: −6.96 to −1.01; *p* = 0.0086), and (SMD = −2.71, 95% CI: −5.12 to −0.30; *p* = 0.0275) respectively, while LOCTR, and two-port ECTR showed similar time to return to work compared to COCTR. Heterogeneity analyses indicated a substantial level of variability in treatment effects (I^2^ = 98.1%, *p* < 0.0001), indicating significant variation in treatment effects both within and between the study designs (ESM. 7).

### Results of operation time

The analysis comparing operational time across different treatments and COCTR revealed that DTT, LOCTR, one-port ECTR, and two-port ECTR displayed a non-significant mean difference compared to COCTR. Heterogeneity analyses indicated a high level of variability in treatment effects (I^2^ = 99.1%, *p* < 0.0001), indicating significant variation in treatment effects both within and between the study designs (ESM. 8).

### Results of adverse events

The analysis of adverse events revealed that DTT is associated with the lowest risk of adverse events compared to Conventional open release (OR = 0.05, 95% CI: 0.01 to 0.42; *p* = 0.01). In contrast, One Port Endoscopic release, Two Port Endoscopic release, and US-guided release showed similar rates of adverse events compared to COCTR. Regarding heterogeneity, the analysis demonstrated low overall inconsistency (I^2^ = 2.8%, 95%, *p* = 0.36), further supporting the overall consistency of the findings (ESM. 9).

### Publications bias

The comparison-adjusted funnel plot did not exhibit notable asymmetry, suggesting minimal evidence of publication bias across the included studies. Additionally, Egger's regression test yielded a non-significant p-value of 0.7897, indicating no statistically significant asymmetry. These results collectively suggest that publication bias is unlikely to have substantially influenced the outcomes of our network meta-analysis (ESM. 11).

### Confidence in network estimates (CINeMA)

The CINeMA evaluation for the primary outcomes is summarized in ESM 10. For BCTQS at 3 months, the confidence in estimates was moderate for one-port ECTR vs COCTR, low for mOCTR and two-port ECTR, and very low for CTR-US due to concerns in indirectness, risk of bias, and imprecision. For pain scores, confidence was rated low for comparisons involving one- and two-port ECTR, and very low for CTR-US, primarily due to imprecision and study limitations. Overall, the evidence for endoscopic techniques remains supportive, but should be interpreted considering the underlying quality of included studies.

## Discussion

While conservative management may offer temporary symptom relief in early or mild cases of carpal tunnel syndrome, surgical intervention remains the mainstay for patients with persistent or severe symptoms. Our network meta-analysis offers a comprehensive comparative evaluation of seven surgical procedures, providing an understanding of their relative effectiveness in symptom relief, functional recovery, and complication rates; thus filling a critical knowledge gap in the clinical decision-making process [6, 8, 54]. To our knowledge, this is the first NMA to compare all CTR procedures with 32 studies involving 3128 patients with a total of 3240 hands.

Our NMA results regarding symptom severity and functional status showed that ECTR has the best relative improvement in either one-port or two-port techniques except for the two-point discrimination outcome where LOCTR has the best results. The one-port technique was superior regarding pinch strength, grip strength, and distal motor latency while the two-port technique was superior in BCTQF, BCTQS, and pain score. The most significant improvements occur by the three-month time point except pain relief where endoscopic techniques, especially two-port, show considerable improvement in pain score in the first month. The relative delay in outcomes other than pain relief may be due to the pathophysiological nature of the disease and its reversibility which may be affected by the period from the onset of the disease to operation time, or degree of the nerve affected. Also, mOCTR showed significant results in terms of BCTQS in three-month duration and BCTQF in all durations. Also, there were similarities between all interventions regarding scar tenderness, one-month duration pinch strength, and six-month duration BCTQS. Despite the current data not supporting CTR-US in terms of symptom severity and functional status, it demonstrated the most and only statistically significant odds ratio in patient satisfaction.

Regarding return to work, one-port ECTR also had the statistically significant quicker time required to return to work, DTT had the lowest risk of adverse effects, and there were similarities between all interventions in terms of operational time.

Our results are in line with a systematic review and meta-analysis by Hammert et al. [55] in which mOCTR significantly improved function and pain. However, while we compared mOCTR with other procedures, both ECTR one and two-port were superior to mOCTR. Also, Vasiliadis et al. and Koong et al. [56, 57] agreed that one-port ECTR had a quicker time regarding return to work. While several comparisons achieved statistical significance, we also considered clinical relevance by referencing known Minimal Clinically Important Differences (MCIDs). For the BCTQ Symptom Severity and Functional Scales, an MCID of 0.2–0.5 points has been suggested; for VAS pain, 1.0–1.5 cm is commonly accepted. Accordingly, the observed reductions for one- and two-port ECTR in these outcomes exceeded MCID thresholds, supporting both statistical and clinical significance.

According to a meta-analysis by Koong et al. [57] ECTR either one or two-port has a higher incidence of adverse events and two-port ECTR has diminished scar tenderness in comparison to open and one-port which is in contrast with our findings, which could be explained by their limitations. They also reported that the decision to undergo ECTR is influenced by factors, such as there is a strong association between pre-operative symptoms and the degree of post-operative improvement. Moreover, the pre-operative patient expectations vary among demographic groups. But they agreed with our results regarding operational time.

According to Moya et al. [58] ultrasound in patients with CTS is associated with improvement in DML and represents a reduction in clinical severity which is inconsistent with our findings. However, they compared CTR-US to a sham ultrasound, no treatment, or ultrasound combined with other interventions, moreover, there is no difference in sample size between our NMA and this meta-analysis regarding this outcome.

According to RCTs by Hamed et. al [51], and Vanni et al. [18] DTT showed faster recovery time and less scar tenderness which is inconsistent with our findings, as they had a smaller sample size and fewer arms. However, they are in line with our findings in terms of lower adverse effects.

Our findings showed no statistically significant difference in scar tenderness between LOCTR and COCTR, which contrasts with the results of Jugovac et al. [35] and Wong et al. [30], who reported reduced scar tenderness with LOCTR. These discrepancies may be attributed to differences in incision techniques and follow-up protocols. For instance, Jugovac et al. employed a microscopic-assisted limited palmar incision technique and noted potential confounding from worker’s compensation status, which may have influenced subjective recovery metrics such as tenderness and return to work. Similarly, Wong et al. observed lower early pillar pain with LOCTR, but the ECTR comparator in their study involved a two-portal technique with a proximal entry wound that may have contributed to local discomfort. In contrast, our analysis pooled various LOCTR techniques, which may have diluted any single technique’s effect. Conversely, Cho et al. [39] and Suppaphol et al. [28] support our findings by showing no significant difference in scar tenderness between LOCTR and COCTR, potentially due to the adoption of nerve-sparing or less traumatic tunneling methods in both groups. These variations highlight the influence of surgical approach, patient selection, and follow-up practices on subjective outcomes like scar tenderness, and reinforce the value of aggregate evidence synthesis through NMA.

Our results showed that more than one procedure is shown to be statistically significant better than COCTR in more than one outcome. Also, there is heterogeneity regarding these outcomes. So, it could be explained that there is variability in decision-making between physicians and patients as it may vary depending on the unique characteristics of each patient’s condition.

## Strengths and limitations

Our study is the first NMA in this area, also our study utilized a rigorous systematic review and network meta-analysis approach following PRISMA guidelines including only high-quality randomized controlled trials (RCTs) eliminating any inherent biases often found in observational studies, which enhanced the strengths of findings and clear presentation of the findings, providing significant visions into the effectiveness of each technique. The inclusion of this network meta-analysis allowed for a comprehensive comparison of multiple techniques for carpal tunnel release exploring their safety and efficacy over multiple periods, to determine the point at which the effect of each technique starts to appear compared to the standard method of release. Nevertheless, we have encountered some limitations in our study starting with the presence of a high risk of bias in three of the included studies, also the follow-up duration varied widely throughout the studies, ranging from a few days to no more than four years postoperatively. Moreover, significant heterogeneity was observed in some outcomes due to variations in study interventions, participants'demographics, and study contexts in the included RCTs as well as the variation in the number of patients across the included RCTs. This network meta-analysis (NMA) has several limitations that should be considered. We conducted a sensitivity analysis after removing the high-risk-of-bias studies, but that did not resolve the heterogeneity and due to limited studies per subgroup and inconsistency in covariate reporting, subgroup or meta-regression analyses were not feasible. Variability in study designs, patient populations, surgical techniques, and follow-up durations may have contributed to heterogeneity, potentially affecting the comparability of results. Some studies exhibited risks of bias, including selection and measurement bias, as well as missing data, which could impact the reliability of findings. Most included studies focused on short- to mid-term outcomes, with insufficient data on long-term efficacy, recurrence rates, and complications. Differences in outcome measurement and reporting further complicate direct comparisons between procedures. Lastly, the generalizability of findings may be limited, as factors such as patient comorbidities, disease severity, and surgeon expertise could influence treatment outcomes.the heterogeneity in these specific outcomes such as scar tenderness, grip strength, and pinch strength is likely due to clinical and methodological variation across studies, including differences in surgical technique standardization, postoperative rehabilitation protocols, hand dominance, measurement tools, and the subjective nature of scar tenderness and variation in strength testing methods may further contribute to inconsistency in these findings.

## Conclusion

ECTR has the best relative improvement in either one-port or two-port techniques regarding symptom severity and functional status. One-port ECTR was superior regarding pinch strength, grip strength, and distal motor latency while the two-port technique was superior in BCTQF, BCTQS, and pain score. Also, one-port ECTR has a quicker time required to return to work, While CTR-US was the best in patient satisfaction, DTT had the lowest risk of adverse effects, and there were similarities between all interventions in terms of scar tenderness and operational time. Patients’ preferences would help in decision-making. Future studies should explore individualized treatment procedures tailored to patient-specific factors such as anatomical considerations.

## Supplementary Information

Below is the link to the electronic supplementary material.

Supplementary file1 (PDF 3161 KB)

## Data Availability

No datasets were generated or analysed during the current study.
