# Supplementary material for: Comparative assessment of seven surgical procedures in Carpal Tunnel Syndrome: a network meta-analysis empowering physician-patient decision-making
Source: Neurosurg Rev. 2025 Jun 5;48(1):486. doi: 10.1007/s10143-025-03587-x (PMC12141404; doi:10.1007/s10143-025-03587-x)
Supplement: Supplementary file 1 — Supplementary file1 (PDF 3161 KB) [file 10143_2025_3587_MOESM1_ESM.pdf]

Table of Contents

1. Title page....3

Authors....3

Affiliations....3

Corresponding author information:....3

2. (ESM. 2)....4

2.1 For BCTQS One Month....4

2.2 For BCTQS Six Months....4

2.3 For Pain (Vas score) One Month....5

3. (ESM. 3)....5

3.1 For Two-Point Discrimination at Three Months....5

4. (ESM. 4)....6

4.1 For Distal Motor Latency at Three Months....6

5. (ESM. 5)....6

5.1 for BCTQF at one month....6

5.2 for BCTQF at Six Months....7

5.3 for Grip Strength at One Month....7

5.4 for Grip Strength at Six Months....8

5.5 for Pinch Strength at One Month....8

5.6 for Pinch Strength at Six Months....9

6. (ESM. 6)....9

6.1 For Patient Satisfaction....9

7. (ESM. 7)....10

7.1 For Return to Work....10

8. (ESM. 8)....10

8.1 For Operation Time....10

9. (ESM. 9)....11

7.1 For Adverse Events....11

10. (ESM. 10)....11

10.1 League Tables:....11

Abbreviations: 11
Pinch Strength: 11
Grip Strength: 12
Boston Carpal Tunnel Questionnaire Functional Status Scale (BCTQF): 12
Boston Carpal Tunnel Questionnaire Symptom Severity Scale (BCTQS): 12
Two-Point Discrimination: 13
Distal Motor Latency (DML): 13
Pain Score: 13
Scar Tenderness: 14
Patient Satisfaction: 14
Return to Work: 14
Operational Time: 14
Adverse Events: 14
11. (ESM. 11) 15
11.1 Publication bias 15

1. Title page

Comparative Assessment of Seven Surgical Procedures in Carpal Tunnel Syndrome: A Network Meta-Analysis Empowering Physician-patient Decision-Making

Authors

Amr Elrosasy[1]† – Mahmoud Diaa Hindawi[2,3]† – Qasi Najah[3,4] – Mohamed Abo Zeid[3,5] – Asem Ahmed Ghalwash[2,3] – Rashad G. Mohamad[2,6] – Eslam Afifi[3,7] – Abdallah Bani-Salameh[3,8] – Hatem Eldeeb[2,3] – Nereen Almosilhy[3,9] – Mohamed Ahmed Shahen[3,10] – Fatma Ahmed Monib[3,11] – Yousef Hawas[3,5]

† Equal contributions as First Authors

Affiliations

1. Faculty of Medicine, Cairo University, Cairo, Egypt
2. Faculty of Medicine, Al-Azhar University, Cairo, Egypt
3. Medical Research Group of Egypt, Negida Academy, Arlington, Massachusetts, USA.
4. Faculty of Medicine, Elmergib University, Alkhums, Libya
5. Faculty of Medicine, Tanta University, Tanta, Egypt
6. Mansoura Manchester Program for Medical Education, Faculty of Medicine, Mansoura University, Mansoura, Egypt
7. Faculty of Medicine, Benha University, Benha, Egypt
8. Faculty Of Medicine, Jordan University of Science & Technology, Jordan
9. Department of Pharmacology and Toxicology, Faculty of Pharmacy, Tanta University, Tanta, Egypt
10. Faculty of Medicine, Suez Canal University, Ismailia, Egypt.
11. Faculty of Medicine, Assiut University, Assiut, Egypt.

Corresponding author information: Amr Elrosasy

Faculty of Medicine Cairo University, Cairo, Egypt.

Email: 10912022103193@stud.cu.edu.eg

ORCID: https://orcid.org/0000-0002-5592-3908

2. (ESM. 2)

2.1 For BCTQS One Month

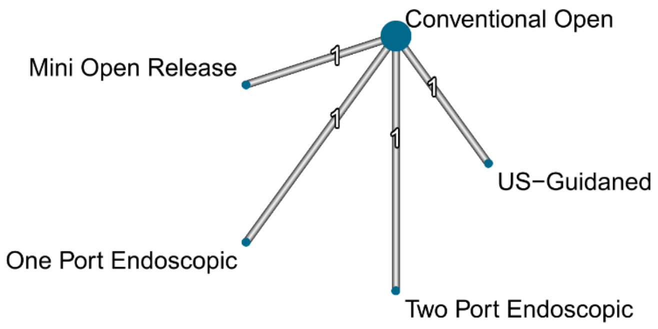

Network plots representing evidence directly are used in NMA. Nodes represent the procedures and edges represent the direct comparisons. Node size and line thickness are proportional to the number of papers providing direct evidence

2.2 For BCTQS Six Months

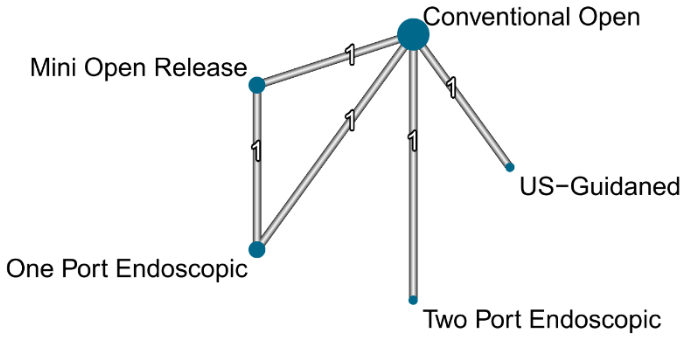

Network plots representing evidence directly are used in NMA. Nodes represent the procedures and edges represent the direct comparisons. Node size and line thickness are proportional to the number of papers providing direct evidence

2.3 For Pain (Vas score) One Month

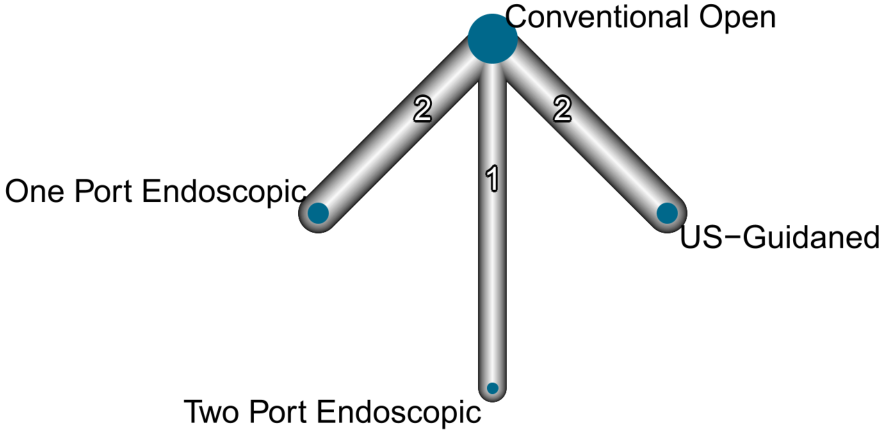

Network plots representing evidence directly are used in NMA. Nodes represent the procedures and edges represent the direct comparisons. Node size and line thickness are proportional to the number of papers providing direct evidence

3. (ESM. 3)

3.1 For Two-Point Discrimination at Three Months

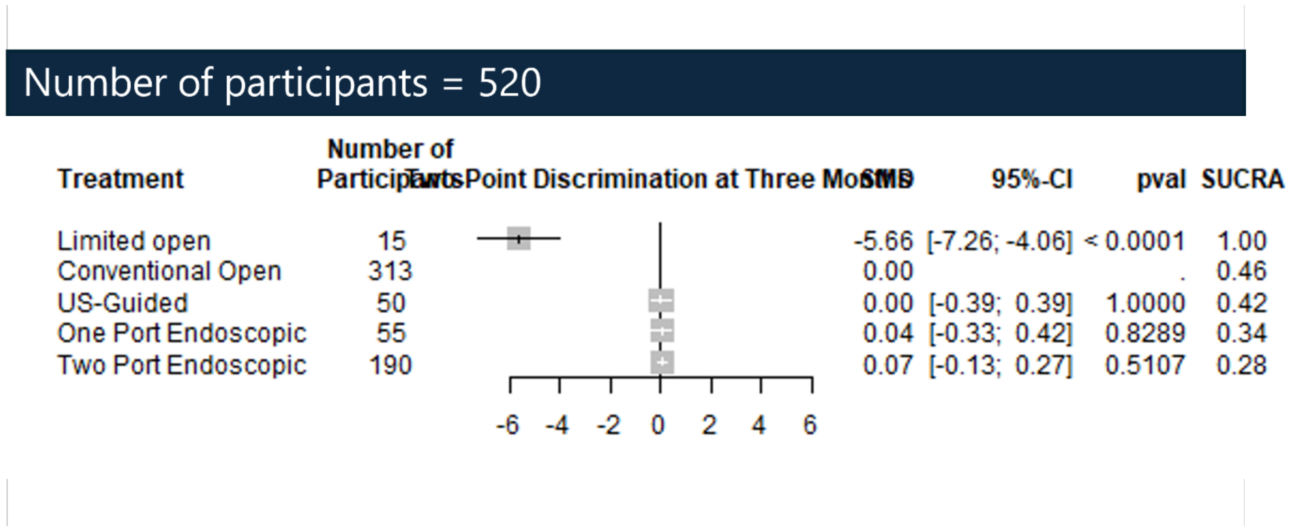

Forest plot with mean difference (MD), confidence interval (CI), and standard deviation (SD)

4. (ESM. 4)

4.1 For Distal Motor Latency at Three Months

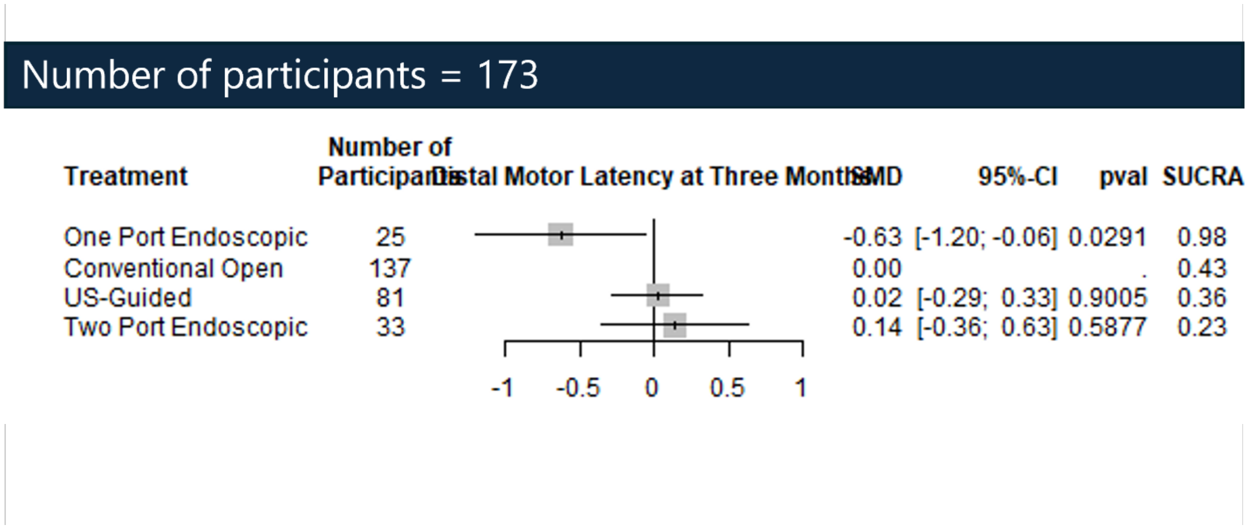

Forest plot with mean difference (MD), confidence interval (CI), and standard deviation (SD)

5. (ESM. 5)

5.1 for BCTQF at one month

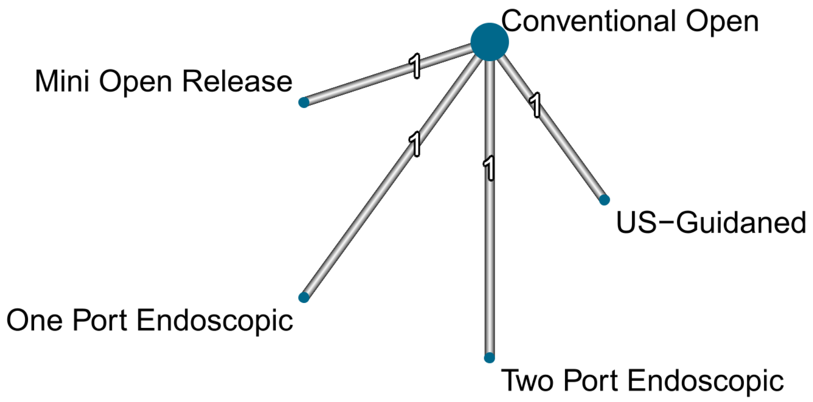

Network plots representing evidence directly are used in NMA. Nodes represent the procedures and edges represent the direct comparisons. Node size and line thickness are proportional to the number of papers providing direct evidence

5.2 for BCTQF at Six Months

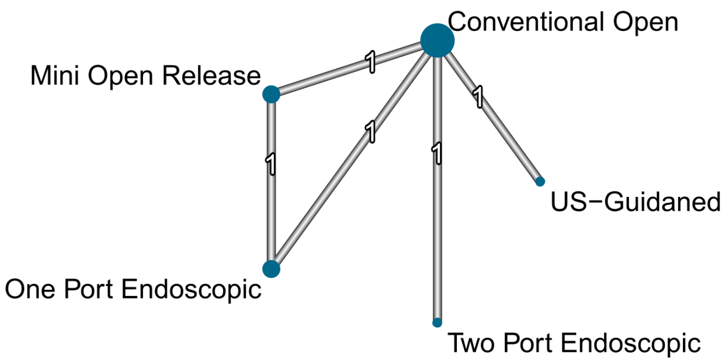

Network plots representing evidence directly are used in NMA. Nodes represent the procedures and edges represent the direct comparisons. Node size and line thickness are proportional to the number of papers providing direct evidence

5.3 for Grip Strength at One Month

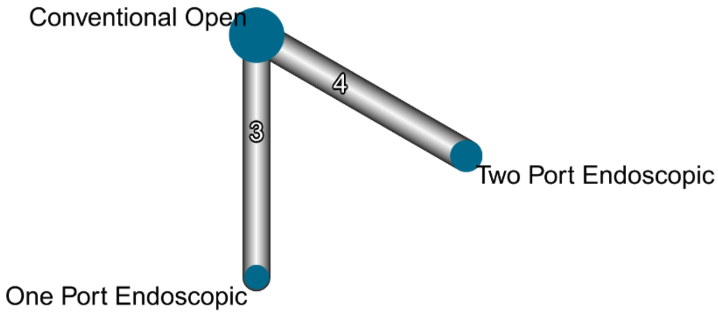

Network plots representing evidence directly are used in NMA. Nodes represent the procedures and edges represent the direct comparisons. Node size and line thickness are proportional to the number of papers providing direct evidence

5.4 for Grip Strength at Six Months

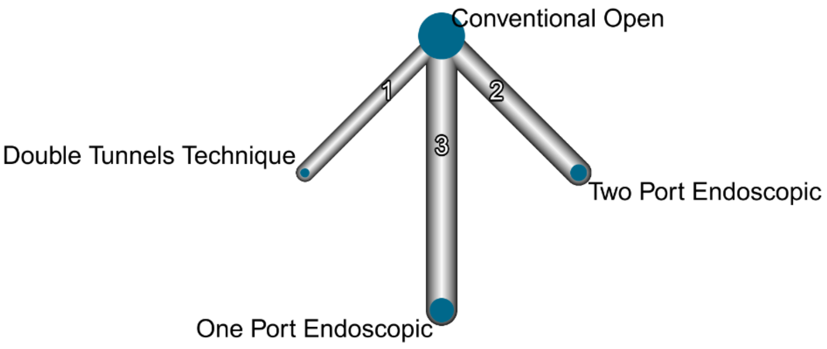

Network plots representing evidence directly are used in NMA. Nodes represent the procedures and edges represent the direct comparisons. Node size and line thickness are proportional to the number of papers providing direct evidence

5.5 for Pinch Strength at One Month

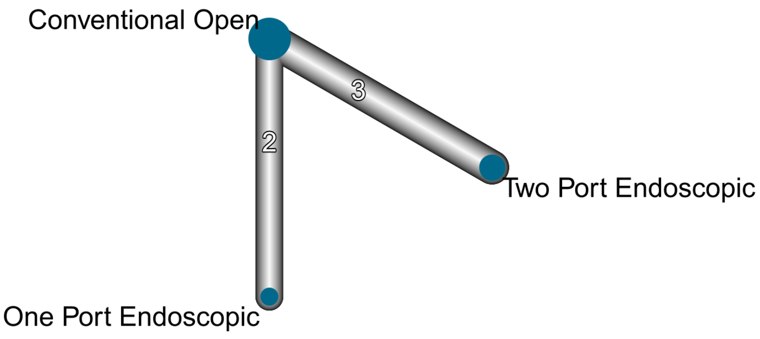

Network plots representing evidence directly are used in NMA. Nodes represent the procedures and edges represent the direct comparisons. Node size and line thickness are proportional to the number of papers providing direct evidence

5.6 for Pinch Strength at Six Months

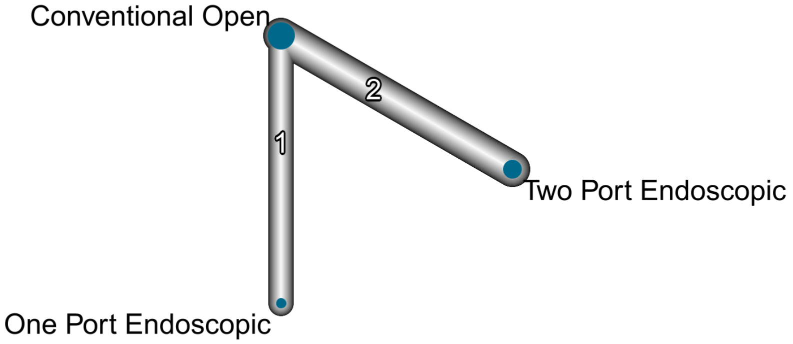

Network plots representing evidence directly are used in NMA. Nodes represent the procedures and edges represent the direct comparisons. Node size and line thickness are proportional to the number of papers providing direct evidence

6. (ESM. 6)

6.1 For Patient Satisfaction

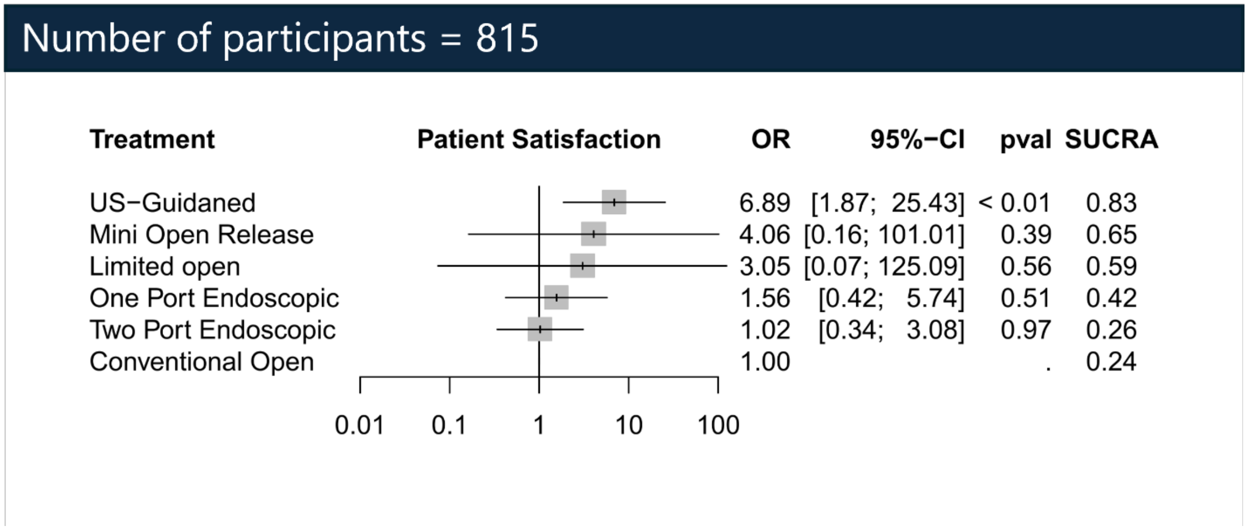

Forest plot with odds ratio (OR), confidence interval (CI), and standard deviation (SD)

7. (ESM. 7)

7.1 For Return to Work

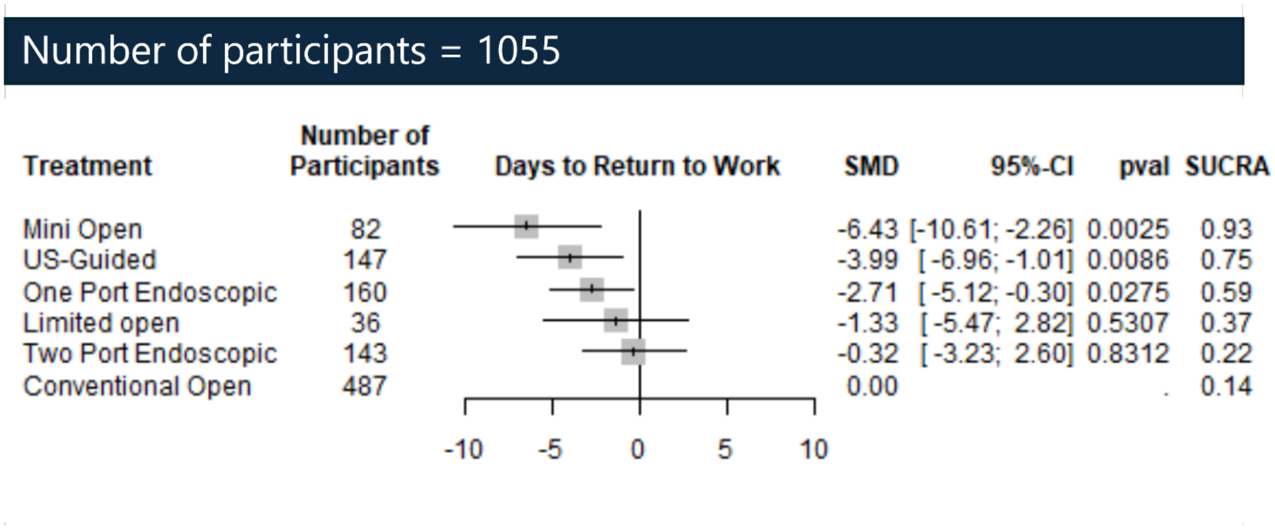

Forest plot with mean difference (MD), confidence interval (CI), and standard deviation (SD)

8. (ESM. 8)

8.1 For Operation Time

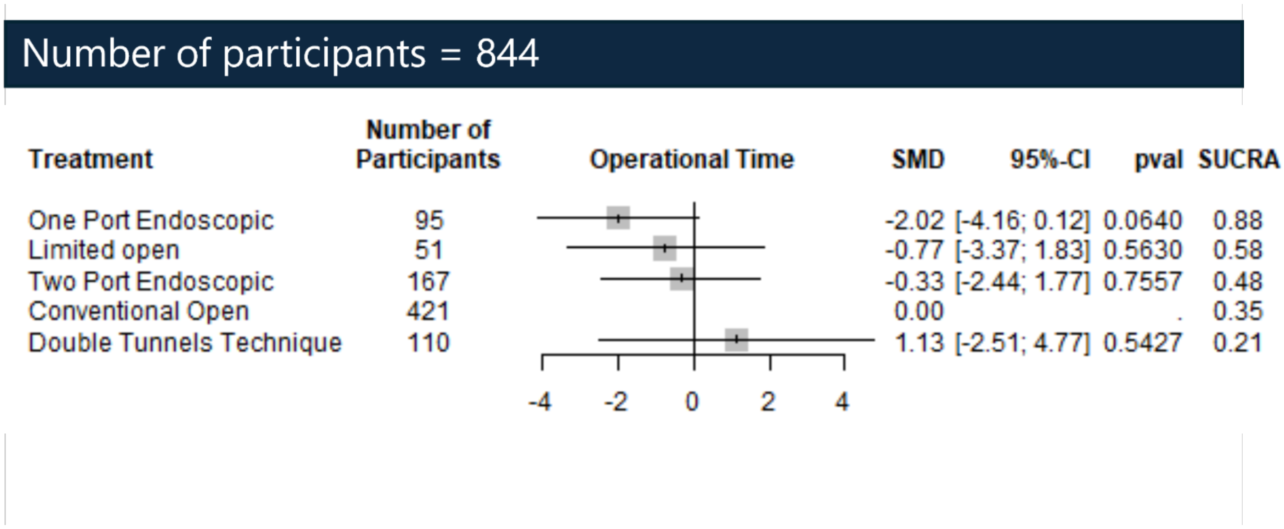

Forest plot with mean difference (MD), confidence interval (CI), and standard deviation (SD)

9. (ESM. 9)

7.1 For Adverse Events

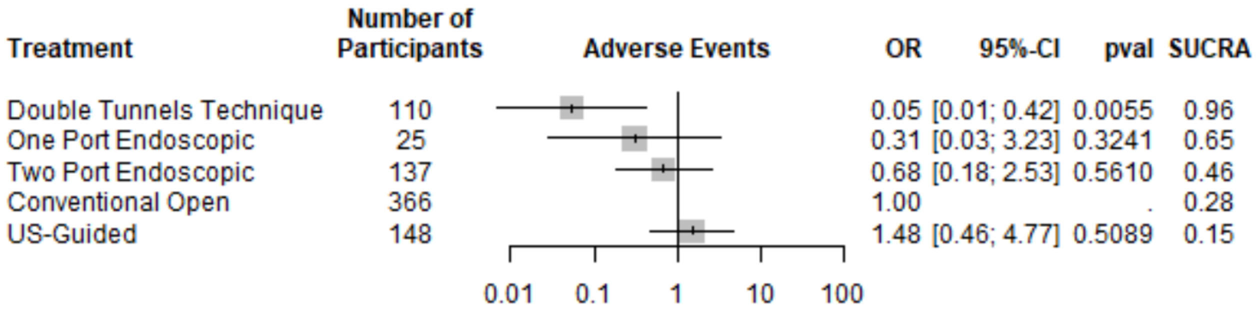

Forest plot with odds ratio (OR), confidence interval (CI), and standard deviation (SD)

10. (ESM. 10)

10.1 League Tables:

Abbreviations: Conventional Open: CO, Double Tunnels Technique: DTT, Limited open: LO, Mini Open: MO, One Port Endoscopic: OPE, Two Port Endoscopic: TPE, US-Guided: USG.

Pinch Strength:

**Pinch Strength at one month**		
CO	1.8 (-3.6; 7.1)	0.9 (-4.2; 6.0)
1.8 (-3.6; 7.1)	OPE	.
0.9 (-4.2; 6.0)	-0.9 (-8.3; 6.5)	TPE
**Pinch Strength at three months**		
CO	-1.2 (-1.9; -0.4)	0.1 (-0.6; 0.7)
-1.2 (-1.9; -0.4)	OPE	.
0.1 (-0.6; 0.7)	1.2 (0.2; 2.2)	TPE
**Pinch Strength at six months**		
CO	-1.5 (-2.0; -1.0)	1.9 (-2.4; 6.2)
-1.5 (-2.0; -1.0)	OPE	.
1.9 (-2.4; 6.2)	3.4 (-0.9; 7.7)	TPE

Grip Strength:

**Grip Strength at one month**			
CO	-4.1 (-7.1; -1.1)	2.1 (-1.0; 5.2)	
-4.1 (-7.1; -1.1)	OPE	.	
2.1 (-1.0; 5.2)	6.2 (1.8; 10.5)	TPE	
**Grip Strength three months**			
CO	-4.0 (-14.2; 6.2)	-4.4 (-7.3; -1.5)	-0.6 (-3.5; 2.4)
-4.0 (-14.2; 6.2)	DTT	.	.
-4.4 (-7.3; -1.5)	-0.4 (-11.0; 10.2)	OPE	.
-0.6 (-3.5; 2.4)	3.4 (-7.2; 14.0)	3.8 (-0.3; 7.9)	TPE
**Grip Strength at six moths**			
CO	-5.0 (-16.5; 6.5)	-4.2 (-7.2; -1.2)	0.1 (-5.4; 5.5)
-5.0 (-16.5; 6.5)	DTT	.	.
-4.2 (-7.2; -1.2)	0.8 (-11.1; 12.6)	OPE	.
0.1 (-5.4; 5.5)	5.1 (-7.6; 17.8)	4.3 (-1.9; 10.5)	TPE

Boston Carpal Tunnel Questionnaire Functional Status Scale (BCTQF):

**BCTQF at one month**				
CO	0.4 (0.2; 0.6)	0.2 (0.1; 0.2)	5.5 (2.9; 8.1)	0.1 (-0.2; 0.4)
0.4 (0.2; 0.6)	MO	.	.	.
0.2 (0.1; 0.2)	-0.2 (-0.4; -0.1)	OPE	.	.
5.5 (2.9; 8.1)	5.1 (2.5; 7.7)	5.3 (2.7; 8.0)	TPE	.
0.1 (-0.2; 0.4)	-0.3 (-0.6; 0.0)	-0.1 (-0.3; 0.2)	-5.4 (-8.0; -2.8)	USG
**BCTQF at three months**				
CO	0.2 (0.1; 0.3)	0.2 (0.1; 0.2)	4.5 (1.9; 7.1)	0.1 (-0.2; 0.3)
0.2 (0.1; 0.3)	MO	0.0 (-0.3; 0.3)	.	.
0.2 (0.1; 0.2)	-0.0 (-0.2; 0.1)	OPE	.	.
4.5 (1.9; 7.1)	4.3 (1.7; 7.0)	4.3 (1.7; 7.0)	TPE	.
0.1 (-0.2; 0.3)	-0.1 (-0.4; 0.1)	-0.1 (-0.3; 0.2)	-4.4 (-7.1; -1.8)	USG
**BCTQF at six months**				
CO	0.4 (0.1; 0.7)	0.1 (0.0; 0.1)	2.8 (0.1; 5.5)	0.1 (-0.3; 0.5)
0.3 (0.1; 0.6)	MO	-0.1 (-0.6; 0.4)	.	.
0.1 (0.0; 0.1)	-0.3 (-0.5; -0.1)	OPE	.	.
2.8 (0.1; 5.5)	2.5 (-0.3; 5.2)	2.7 (0.0; 5.4)	TPE	.
0.1 (-0.3; 0.5)	-0.2 (-0.7; 0.2)	0.0 (-0.4; 0.5)	-2.7 (-5.4; 0.0)	USG

Boston Carpal Tunnel Questionnaire Symptom Severity Scale (BCTQS):

**BCTQS at one month**				
CO	0.3 (0.1; 0.5)	0.1 (0.1; 0.1)	3.9 (-0.4; 8.2)	0.1 (-0.2; 0.4)
0.3 (0.1; 0.5)	MO	.	.	.

0.1 (0.1; 0.1)	-0.2 (-0.4; 0.0)	OPE	.	.
3.9 (-0.4; 8.2)	3.6 (-0.7; 7.9)	3.8 (-0.5; 8.1)	TPE	.
0.1 (-0.2; 0.4)	-0.2 (-0.5; 0.1)	-0.0 (-0.3; 0.3)	-3.8 (-8.1; 0.5)	USG
**BCTQS at three months**				
CO	0.2 (0.1; 0.3)	0.2 (0.1; 0.2)	47.4 (43.7; 51.1)	0.3 (0.1; 0.5)
0.2 (0.1; 0.3)	MO	0.0 (-0.3; 0.3)	.	.
0.2 (0.1; 0.2)	-0.0 (-0.1; 0.1)	OPE	.	.
47.4 (43.7; 51.1)	47.2 (43.5; 50.9)	47.2 (43.5; 50.9)	TPE	.
0.3 (0.1; 0.5)	0.1 (-0.2; 0.3)	0.1 (-0.1; 0.3)	-47.1 (-50.8; -43.4)	USG
**BCTQS at six months**				
CO	0.4 (0.0; 0.8)	0.1 (-0.2; 0.3)	1.0 (-1.6; 3.6)	0.2 (-0.2; 0.6)
0.3 (0.0; 0.6)	MO	0.0 (-0.5; 0.5)	.	.
0.1 (-0.1; 0.3)	-0.2 (-0.5; 0.2)	OPE	.	.
1.0 (-1.6; 3.6)	0.7 (-1.9; 3.3)	0.9 (-1.7; 3.5)	TPE	.
0.2 (-0.2; 0.6)	-0.1 (-0.6; 0.4)	0.1 (-0.4; 0.5)	-0.8 (-3.4; 1.8)	USG

Two-Point Discrimination:

CO	-0.3 (-1.7; 1.1)	0.8 (0.5; 1.1)	-0.0 (-0.4; 0.3)
-0.3 (-1.7; 1.1)	LO	.	.
0.8 (0.5; 1.1)	1.1 (-0.3; 2.6)	OPE	.
-0.0 (-0.4; 0.3)	0.3 (-1.2; 1.7)	-0.9 (-1.3; -0.4)	TPE

Distal Motor Latency (DML):

CO	0.1 (0.0; 0.2)	-0.2 (-0.9; 0.5)	0.3 (-0.7; 1.3)
0.1 (0.0; 0.2)	OPE	.	.
-0.2 (-0.9; 0.5)	-0.3 (-1.0; 0.4)	TPE	.
0.3 (-0.7; 1.3)	0.2 (-0.8; 1.2)	0.5 (-0.8; 1.7)	USG

Pain Score:

**Pain Score at one month**			
CO	0.5 (-0.2; 1.2)	5.4 (4.2; 6.6)	0.1 (-0.6; 0.9)
0.5 (-0.2; 1.2)	OPE	.	.
5.4 (4.2; 6.6)	4.9 (3.5; 6.4)	TPE	.
0.1 (-0.6; 0.9)	-0.3 (-1.4; 0.7)	-5.3 (-6.7; -3.9)	USG
**Pain Score at three months**			
CO	-0.1 (-0.5; 0.4)	1.0 (0.2; 1.7)	0.1 (-0.5; 0.7)
-0.1 (-0.5; 0.4)	OPE	.	.
1.0 (0.2; 1.7)	1.0 (0.2; 1.9)	TPE	.
0.1 (-0.5; 0.7)	0.2 (-0.6; 0.9)	-0.9 (-1.8; 0.1)	USG

Scar Tenderness:

CO	0.7 (0; 26.9)	3.1 (0; 386.2)	.	6.0 (0.0; 1021.7)	2.9 (0.1; 102.2)
0.7 (0; 26.9)	DTT	.	.	.	.
3.1 (0; 386.2)	4.4 (0; 1829.5)	LO	1.3 (0; 144)	.	.
4.1 (0.; 3446)	5.8 (0; 12111)	1.3 (0; 144)	MO	.	.
6.0 (0; 1021.7)	8.4 (0; 4545.3)	1.9 (0.; 217)	1.4 (0; 685)	OPE	.
2.9 (0.1; 102)	4.0 (0; 655.7)	0.9 (0; 36.)	0.7 (0; 1400)	0.5 (0; 249.2)	TPE

Patient Satisfaction:

CO	.	0.2 (0.0; 6.1)	0.6 (0.2; 2.4)	1.0 (0.3; 2.9)	0.1 (0.0; 0.5)
0.3 (0.0; 13.5)	LO	0.8 (0.1; 4.8)	.	.	.
0.2 (0.0; 6.1)	0.7 (0.1; 4.8)	MO	.	.	.
0.6 (0.2; 2.4)	2.0 (0.0; 100.5)	2.6 (0.1; 83.8)	OPE	.	.
1.0 (0.3; 2.9)	3.0 (0.1; 143.5)	4.0 (0.1; 118.6)	1.5 (0.3; 8.4)	TPE	.
0.1 (0.0; 0.5)	0.4 (0.0; 22.7)	0.6 (0.0; 18.9)	0.2 (0.0; 1.4)	0.1 (0.0; 0.8)	USG

Return to Work:

CO	15.0 (-7.7; 37.7)	9.1 (-13.0; 31.3)	15.8 (1.9; 29.6)	4.0 (-11.8; 19.7)	11.3 (-4.3; 27.0)
15.0 (-7.7; 37.7)	LO	.	.	.	.
9.1 (-13.0; 31.3)	-5.9 (-37.5; 25.8)	MO	.	.	.
15.8 (1.9; 29.6)	0.8 (-25.8; 27.4)	6.6 (-19.5; 32.7)	OPE	.	.
4.0 (-11.8; 19.7)	-11.0 (-38.7; 16.6)	-5.2 (-32.4; 22.0)	-11.8 (-32.8; 9.2)	TPE	.
11.3 (-4.3; 27.0)	-3.7 (-31.3; 23.9)	2.2 (-24.9; 29.3)	-4.4 (-25.3; 16.5)	7.4 (-14.9; 29.6)	USG

Operational Time:

CO	-4.3 (-20.0; 11.4)	1.0 (-10.2; 12.1)	7.1 (-2.2; 16.4)	1.2 (-7.9; 10.3)
-4.3 (-20.0; 11.4)	DTT	.	.	.
1.0 (-10.2; 12.1)	5.3 (-14.0; 24.5)	LO	.	.
7.1 (-2.2; 16.4)	11.4 (-6.8; 29.6)	6.2 (-8.3; 20.6)	OPE	.
1.2 (-7.9; 10.3)	5.5 (-12.6; 23.7)	0.3 (-14.1; 14.6)	-5.9 (-18.9; 7.1)	TPE

Adverse Events:

CO	18.6 (2.4; 146.0)	3.3 (0.3; 34.5)	1.5 (0.4; 5.5)	0.7 (0.2; 2.2)
18.6 (2.4; 146.0)	DTT	.	.	.
3.3 (0.3; 34.5)	0.2 (0.0; 4.0)	OPE	.	.
1.5 (0.4; 5.5)	0.1 (0.0; 0.9)	0.5 (0.0; 6.7)	TPE	.

0.7 (0.2; 2.2)	0.0 (0.0; 0.4)	0.2 (0.0; 2.9)	0.5 (0.1; 2.7)	USG

11. (ESM. 11)

11.1 Publication bias

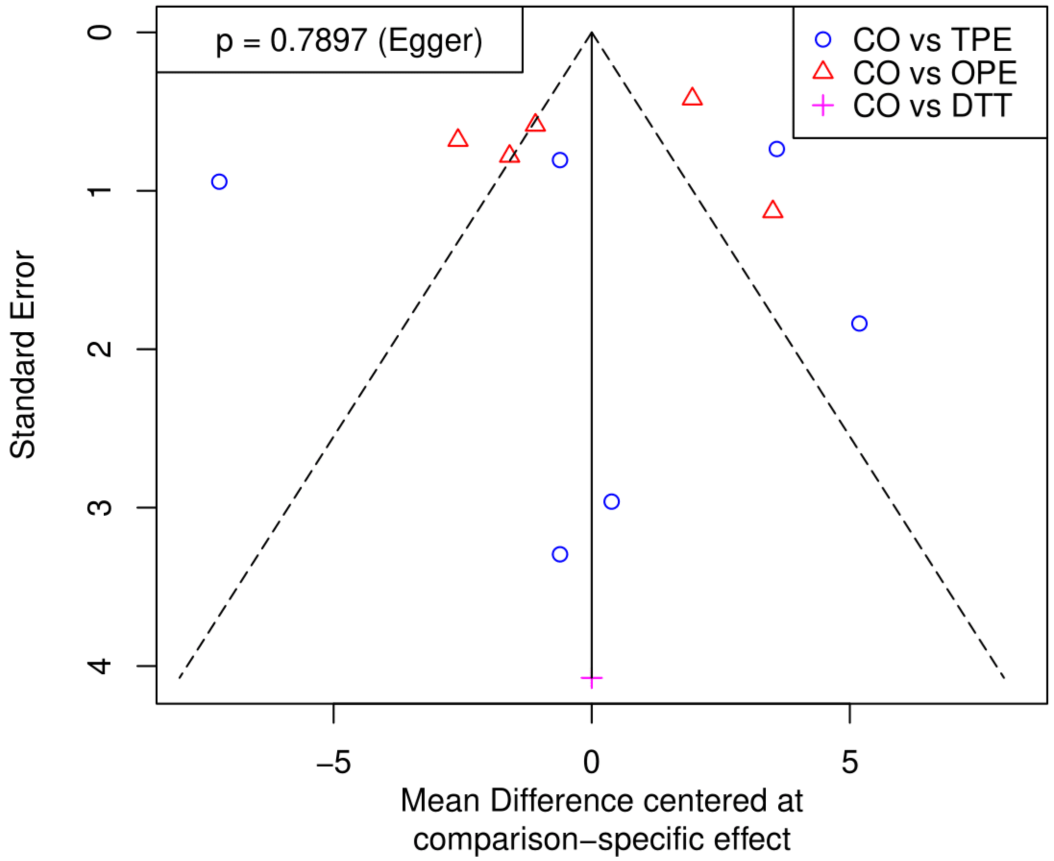

12. (ESM.12)

CINeMA Table – Symptom Severity (BCTQS at 3 Months)

Comparison	Within-study bias	Reporting bias	Indirectness	Imprecision	Heterogeneity	Incoherence	Overall Confidence
One-port ECTR vs COCTR	Some concerns	No concerns	No concerns	No concerns	No concerns	No concerns	**Moderate**
Two-port ECTR vs COCTR	Major concerns	No concerns	Some concerns	No concerns	Some concerns	Some concerns	**Low**
mOCTR vs COCTR	Some concerns	No concerns	No concerns	Some concerns	No concerns	No concerns	**Low**
CTR-US vs COCTR	Some concerns	Some concerns	Some concerns	Major concerns	No concerns	No concerns	**Very low**

13. (ESM.13)

CINeMA Table – Pain Score (VAS)

Comparison	Within-study bias	Reporting bias	Indirectness	Imprecision	Heterogeneity	Incoherence	Overall Confidence
Two-port ECTR vs COCTR	Major concerns	No concerns	Some concerns	No concerns	No concerns	No concerns	**Low**
One-port ECTR vs COCTR	Some concerns	No concerns	No concerns	Major concerns	No concerns	No concerns	**Low**
CTR-US vs COCTR	Some concerns	Some concerns	Some concerns	Major concerns	No concerns	No concerns	**Very low**
